# Genetic factors for survival in amyotrophic lateral sclerosis: an integrated approach combining a systematic review, pairwise and network meta-analysis

**DOI:** 10.1186/s12916-022-02411-3

**Published:** 2022-06-27

**Authors:** Wei-Ming Su, Xiao-Jing Gu, Qing-Qing Duan, Zheng Jiang, Xia Gao, Hui-Fang Shang, Yong-Ping Chen

**Affiliations:** 1 Department of Neurology, West China Hospital, Sichuan University, Chengdu, 610041 Sichuan China; 2 Lab of Neurodegenerative Disorders, Institute of Inflammation and Immunology (III), Frontiers Science Center for Disease-Related Molecular Network, West China Hospital, Sichuan University, Chengdu, 610041 Sichuan China; 3 Centre for Rare Diseases, West China Hospital, Sichuan University, Chengdu, 610041 Sichuan China; 4 Department of Geriatrics, Dazhou Central Hospital, Dazhou, Sichuan China

**Keywords:** Amyotrophic lateral sclerosis, Genes, Variants, Modifier, Survival

## Abstract

**Background:**

The time of survival in patients with amyotrophic lateral sclerosis (ALS) varies greatly, and the genetic factors that contribute to the survival of ALS are not well studied. There is a lack of a comprehensive study to elucidate the role of genetic factors in the survival of ALS.

**Methods:**

The published studies were systematically searched and obtained from PubMed, EMBASE, and the Cochrane Library without any language restrictions from inception to Oct 27, 2021. A network meta-analysis for ALS causative/risk genes and a systematic review and pairwise meta-analysis for other genetic modifiers were conducted. The PROSPERO registration number: CRD42022311646.

**Results:**

A total of 29,764 potentially relevant references were identified, and 71 papers were eligible for analysis based on pre-decided criteria, including 35 articles in network meta-analysis for 9 ALS causative/risk genes, 17 articles in pairwise meta-analysis for four genetic modifiers, and 19 articles described in the systematic review. Variants in three genes, including *ATXN2* (HR: 3.6), *C9orf72* (HR: 1.6), and *FUS* (HR:1.8), were associated with short survival of ALS, but such association was not identified in *SOD1*, *TARDBP*, *TBK1*, *NEK1*, *UBQLN2*, and *CCNF*. In addition, *UNC13A* rs12608932 CC genotype and *ZNF521B* rs2275294 C allele also caused a shorter survival of ALS; however, *APOE* ε4 allele and *KIFAP3* rs1541160 did not be found to have any effect on the survival of ALS.

**Conclusions:**

Our study summarized and contrasted evidence for prognostic genetic factors in ALS and would help to understand ALS pathogenesis and guide clinical trials and drug development.

**Supplementary Information:**

The online version contains supplementary material available at 10.1186/s12916-022-02411-3.

## Background

Amyotrophic lateral sclerosis (ALS) is one of the most devastating neurodegenerative diseases, characterized by degeneration of the upper and lower motor neurons. It eventually results in progressive muscle atrophy and death in 3–5 years after disease onset [1]. About 5% to 10% of patients with ALS are present with a family history, called family ALS(FALS), while the remaining cases are sporadic (SALS) [2]. FALS always occurs due to a specific genetic mutation, but genetic causes also have been known to play an important role in SALS [1, 3]. The cumulative number of ALS-related genes has increased rapidly. To date, more than 130 genes/loci are reported to be associated with a risk of ALS [4]. Some of them also were reported to have a disease modification effect, which means they are always linked to a difference in the clinical phenotype of ALS, often survival time.

As we all know, aging, environmental and genetic factors play an essential role in the development of ALS. However, as a rare disease, we still don’t have an excellent strategy for preventing it from developing due to the limited knowledge of its etiology. Hence, more attention has been paid to the associated factors that affect the survival time. In our recent study, twenty-five non-genetic factors associated with ALS survival were identified, such as age at onset, onset site, the time between onset and diagnosis, et al. [5]. However, these non-genetic factors associated with ALS survival could be affected by confounding factors. Therefore, other unbiased methods exploring clinical outcomes are in the ascendant, such as the Mendelian Randomization study, which focuses on the actual causal effect on diseases or their phenotype by applying genetic variants. Till now, the genetic factors that contribute to the survival of ALS are not well studied and remain to be explored. Previous researches have reported that some potential loci may modify the survival of ALS, such as *UNC13A* rs12608932 and *CAMTA1* rs2412208 [6–8]. In addition, the causative ALS genes (*C9orf72*, *SOD1*, *FUS*, *TARDBP*) might modify the disease course as well [9–13]. Yet, due to the limited sample size of patients with rare variants in ALS causative genes, the different genetic backgrounds, or other factors, there are inconsistencies in those results and a lack of a comprehensive review to elucidate the role of genetic factors in the survival of ALS. Consequently, our study tries to clarify the genetic factors that affect the survival of ALS by an integrated approach combining a network meta-analysis (NMA) on ALS causative/risk genes and a pairwise meta-analysis on other modified loci along with a systematic review.

## Methods

Different genetic variants are considered as different interventions in this study, so we employed a NMA following the International Society for Pharmacoepidemiology and Outcomes Research (ISPOR) guidelines [14] and reported it using the Preferred Reporting Items for Systematic Reviews and Meta-Analyses (PRISMA) extension for NMA [15]. In addition, the report of the pairwise meta-analysis for other modified loci was followed by the recommendations of the PRISMA (2009) guidelines [16]. The protocol for this study was registered with PROSPERO, registration number: CRD42022311646.

### Search strategies and selection criteria

The published studies were systematically searched and obtained from PubMed, EMBASE, and the Cochrane Library without any language restrictions, by using the term: “(gene* OR geno* OR variant* OR mutation OR haplotyp* OR polymorphism* OR SNP OR Allel*) AND (prognosis* OR progress* OR survival OR outcome OR mortality OR death) AND ((amyotrophic lateral sclerosis) OR (motor neuron disease) OR (Lou Gehrig’s disease) OR (Gehrig Disease))”. Reference lists of full review articles were also reviewed to search for additional articles. All randomized clinical trials (RCTs), quasi-RCTs, and observational studies were eligible, but no RCTs were identified. The articles were updated to October 27, 2021.

According to previous survival research in ALS, survival was defined as the time between the onset of symptoms and noninvasive ventilation (NIV) for more than 23 hours per day, or tracheostomy or death [5, 17, 18]. The inclusion criteria are as follows: (1) assessed the association between present or absence with different genetic loci and survival time from the onset in patients with ALS; (2) had reported a hazard ratio (HR) and 95% confidential intervals (CI) for patients with genetic mutations or Kaplan-Meier plots from which we can estimate the HR and 95%CI; (3) abstract, reviews, letters without original study, secondary studies and studies that HR or 95%CI was unavailable in papers, excluded from the analysis; and (4) full papers published in English.

### Data collection

First author’s name, year of publication or online, patients’ nation, number of patients, type of disease, diagnosis criteria, age at onset and gender distribution of patients, median survival time, and HR with their 95% CI were extracted. When HR was unavailable directly from the articles, the Kaplan-Meier curve was evaluated by Engauge Digitizer version 12.1, and HRs and 95%CI were estimated using Richard Steven’s excel workbook [19]. Data from multi-arm studies were extracted following the tutorial by B. S. Woods [20]. Extracted data from included studies by two independent reviewers to reduce bias and a third one verified the data to avoid repeat inclusion.

### Appraisal of methodological quality

Only observational studies were included for analysis in this study, and their quality was appraised with the Newcastle-Ottawa Scale (NOS) [21]. As for publication bias, the assessment was conducted only when at least ten studies were available for the same factor by Begg’s test [22]. In addition, to overcome overestimation and mask funnel plot asymmetry induced by some multi-arm studies, we plotted data points corresponding to the study-specific basic parameters (different known ALS gene mutation comparisons with a common comparator). In each study, we used the control group (absence with known ALS gene mutation) as the common comparator, if this was unavailable, we used the same comparator (always, group with *SOD1* mutation) against the remaining groups.

### Synthesis of included studies

HR was used for each dichotomous outcome, and traditional pairwise meta-analyses were performed for studies, which directly compared different groups. And from known ALS causative/risk genes variants, outcome data were pooled using NMA models through the R 4.1.1 software. A network relationship diagram was drawn, among which parameters such as each node and line thickness respectively represent a certain gene’s variants and the researches sizes were considered from the included studies. The model fit was assessed using three criteria based on the deviance and node-based residuals. We evaluated the inconsistency, which means the difference between the pooled direct and indirect evidence of a particular comparison, using inconsistency factors based on a modified back-calculation approach [23]. In addition, we performed the rankogram plots to show the probability of each genetic mutation [24]. The remaining modification loci were pool analyzed under meta-analysis by random-effect model if analyzed in more than two studies (≥3 studies); otherwise they were conducted for systematic review if analyzed in less than three studies (< 3 studies) (including *SMN2* deletion, *SMN1* and *SMN2* copy numbers, *CX3CR1* V249I, *CX3CR1* T280M, haplotype in *CX3CR1*, *ABCC8* rs4148646, *KCNJ11* rs5219, *LXRs* rs2279238, *LXRs* rs7120118, *LXRs* rs35463555, *LXRs* rs2695121, *PRGN* rs9897526, *PRGN* rs34424835, haplotype in *PRGN*, *HTR2B* rs10199752, *STMN2* CA repeat, *BDNF* C270T, *C7* gene rs3792646, *PON1* rs854560, *PON1* rs662, *NIPA1* polyalanine repeat expansions, *SLC11A2* rs407135, *CAMTA1* rs2412208, *GSTP1* rs1695, *CNTF*, *HLA-DRA or HLA-DRB5* rs9268856, rs4623951, *EPHA4* rs6436254, and *IDE* rs139550538*)*. All computations were conducted on R (V4.1.1) package “gemtc,” “rjags,” and STATA/MP 16.0.

## Results

### Literature results

**Fig. 1:**
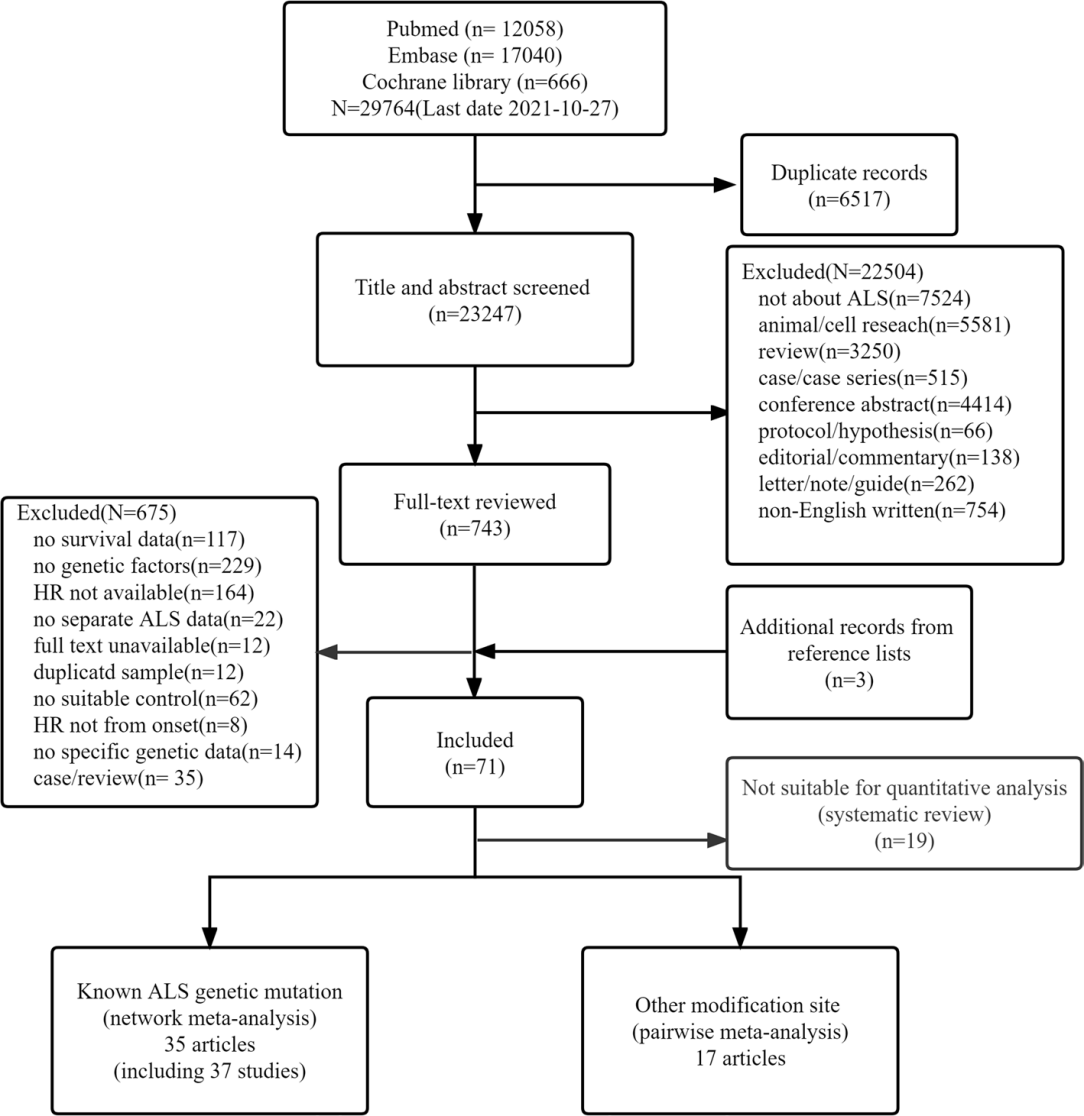
Flow chart for literature selection. ALS, amyotrophic lateral sclerosis

We identified 29,764 potentially relevant references from PubMed, EMBASE, and Cochrane Library and three additional records from reference lists (Fig. 1). Of these records, only 71 articles met the inclusion criteria. Finally, we included 35 articles using network meta-analysis for variants in nine ALS causative/risk genes (Additional file 1: Table S1) [9–13, 25–54], 17 articles using pairwise meta-analysis for four modification loci in ALS-related genes (Additional file 1: Table S2) [6–8, 55–68], and 19 articles using systematic review according to pre-decided criteria (Additional file 1: Table S3) [7, 69–87]. The characteristics of the 71 included trials are summarized in Additional file 1: Table S1-S3 and the quality of 52 articles conducted by network meta-analysis and pairwise meta-analysis was shown in Additional file 1: Table S4**.**

### Variants in nine ALS causative/risk genes by NMA

**Fig. 2:**
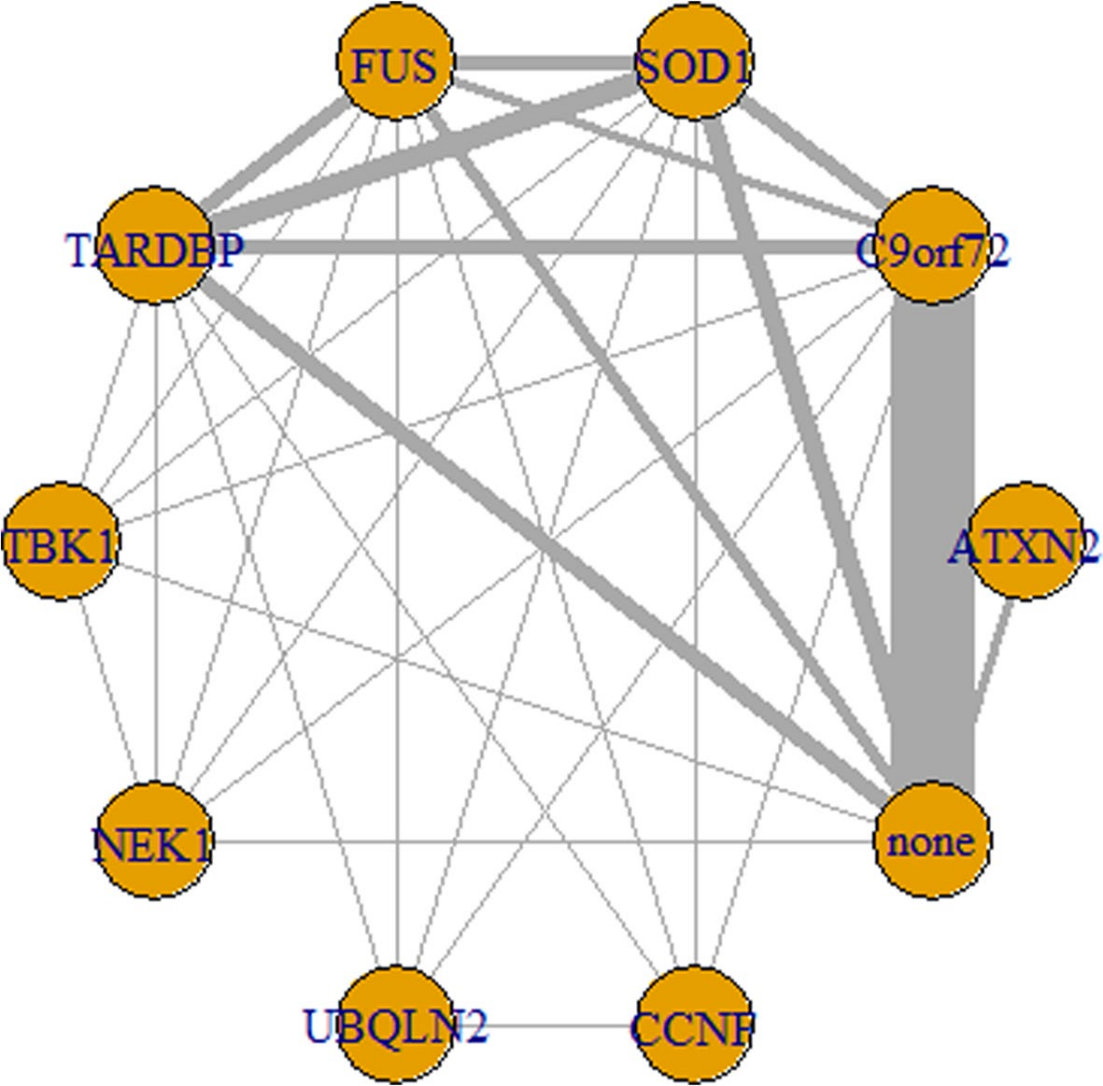
Network of analyzed comparisons. Each circle corresponds to a regimen included in the analysis. “none” means that there were no known ALS genetic variants, and the others mean that there were corresponding genetic variants shown in the circle. Each line represents comparisons between regimens, with the thickness corresponding to the number of within-trial comparisons

**Fig. 3:**
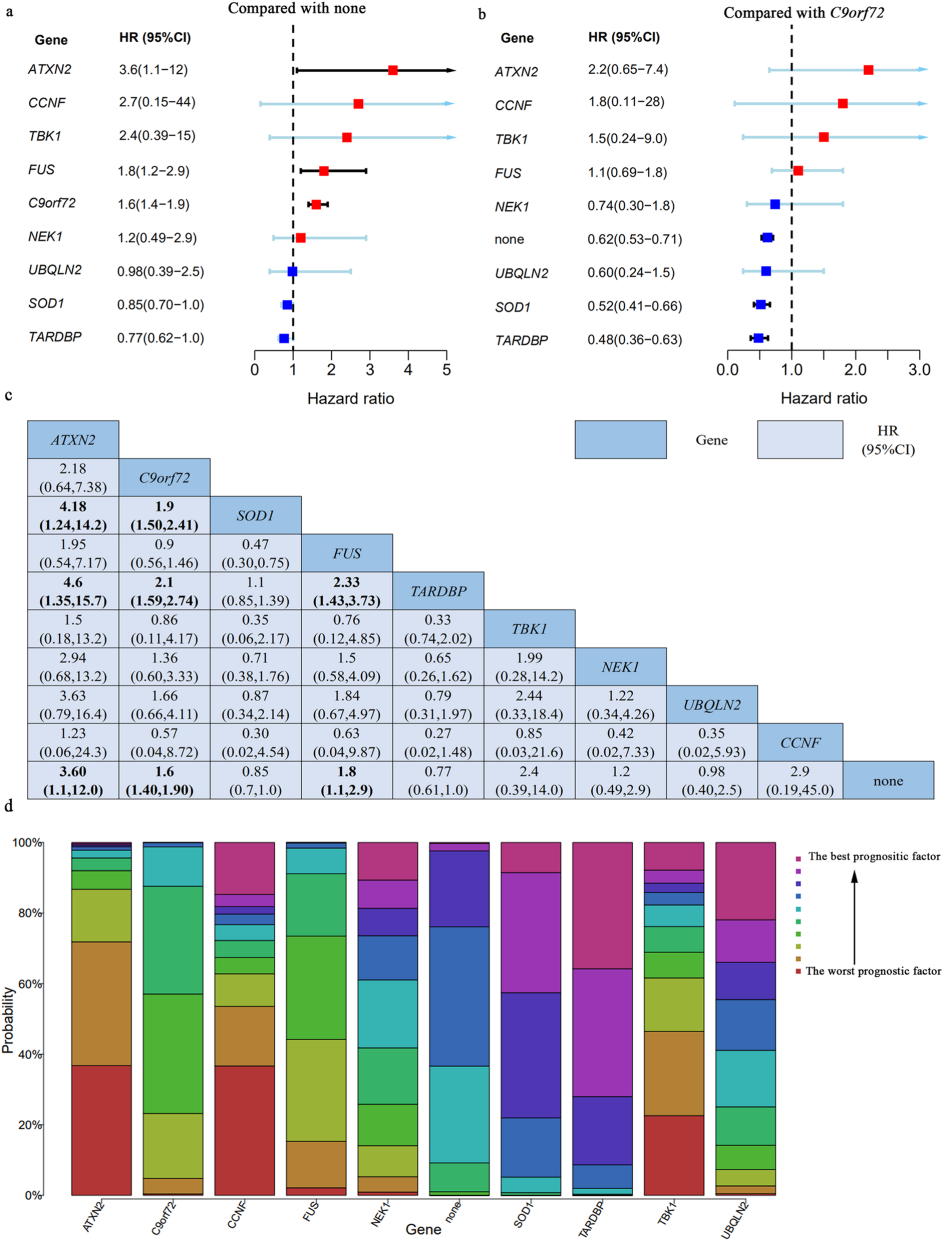
Forest plots of HR for pairwise comparison and rank probabilities for each regimen included in network meta-analysis. **a** Forest plot of HR for each genetic mutation when compared to absence with genetic mutation. **b** Forest plot of HR for absence with genetic mutation and other genetic mutation compared to *C9orf72* repeat expansion. **c** The result of pairwise comparison among these genes. Bold: significative. **d** Rank probabilities among those genes for ALS survival based on the network meta-analysis. CI, confidence interval

A total of 35 articles including 37 trials with nine known ALS causative/risk genes were involved in this network meta-analysis, which is shown in Fig. 2. *C9orf72* was the most frequently investigated regimen with 40 comparisons (Additional file 1: Table S1). It was found that ALS patients carrying *C9orf72* repeat expansion would have a poor prognosis compared to those without known ALS causative/risk genes variants and the HR was 1.6 (95%CI:1.4–1.9). In addition, patients with other two genes variants, *ATXN2* and *FUS*, presented a short survival time compared to those without known ALS causative genes variants (HR:3.6 or 1.8, respectively). However, *SOD1*, *TARDBP*, *TBK1*, *NEK1*, *UBQLN2*, and *CCNF* did not affect the survival of ALS. The detailed features for each gene are shown in Table 1. Compared to *C9orf72* repeats expansion, patients presented with *SOD1* or *TARDBP* variants or without genetic variants seem to possess a better prognosis. However, no difference between *C9orf72* repeats expansion and *FUS* variants and *ATXN2* polyQ were identified. The detailed results are illustrated in Fig. 3.

**Table 1:** The characteristics of genes included in network meta-analysis

Gene	Full name	Location	Category in ALSoD	Proportion of patients with ALS	Common mutations in ALS	Possible pathogenesis	Phenotype	Total HR (95% CI)^a^
*ATXN2*	Ataxin 2	12q24.12	Clinical modifier	<1% in northern European ancestry [88, 89], about 2% in French/French Canadian [90], about 2.5% in Italian [91], < 1% in Turkish [92] and in Chinese [93].	CAG repeat size ≥ 31	neurotoxicity caused by prion-like self-assembly and propagation of mutant or misfold protein [1, 94]	Mainly spinal onset	3.6 (1.1, 12)
*C9orf72*	C9orf72-SMCR8 complex subunit	9p21.2	Definitive ALS gene	FALS 33.7%, SALS 5.1% in European, FALS 2.3%, SALS 0.3% in Asian [95].	GGGGCC hexanucleotide repeat expansion usually > 30, even > 1000	RNA foci-mediated toxicity; dipeptide repeat protein (DPR)-mediated toxicity; and/or reduced levels of C9orf72 protein [96]	Mainly spinal onset, always with FTD	1.6 (1.4, 1.9)
*SOD1*	Superoxide dismutase 1	21q22.11	Definitive ALS gene	FALS 14.8%, SALS 1.2% in European. FALS 30.0%, SALS 1.5% in Asian [95].	A4V, I113T, G41A, A4T, G37R, D90A, D101N, E100G, A140A, D76Y, E21G, H46R, I149T, L106V, L144F, et al	autophagy, mitophagy, and neuroinflammation by mutant or misfold protein aggregates due to decreased SOD1 enzymatic activity [1, 97]	Mainly spinal onset	0.85 (0.70, 1.0)
*FUS*	Fused in sarcoma	16p11.2	Definitive ALS gene	FALS 2.8%, SALS 0.3% in European. FALS 6.4%, SALS 0.9% in Asian [95].	P525L, R495X, R521H, R521C, G504WfsX12, et al	a toxic gain-of-function due to FUS aggregation and/or a loss-of-function resulting from cytoplasmic mis-localization of FUS and subsequent loss of nuclear function [1, 98]	Mainly spinal onset, early-onset	1.8 (1.1, 2.9)
*TARDBP*	TAR DNA binding protein	1p36.22	Definitive ALS gene	FALS 4.2%, SALS 0.8% in European. FALS 1.5%, SALS 0.2% in Asian [95].	A382T, A382T, G294V, G295S, G348C, M337V, et al	neurotoxicity and motor neuron caused by degeneration dysregulation of transposable elements due to TDP-43 loss-of-function [1, 99]	Mainly spinal onset	0.77 (0.61, 1.0)
*TBK1*	TANK binding kinase 1	12q14.2	Definitive ALS gene	About 2.8% of ALS patients [100]	T79del, G272_T331del, 690-713del, et al	autophagy, mitophagy, and neuroinflammation due to decrease Tbk1’s kinase activity [101]	Mainly spinal onset	2.4 (0.39, 14.0)
*NEK1*	NIMA related kinase 1	4q33	definitive ALS gene	About 3% of ALS patients [102]	M545T, R261H, et al	impaired function for DNA damage repair [103]	NA	1.2 (0.49, 2.9)
*UBQLN2*	Ubiquilin 2	Xp11.21	Definitive ALS gene	rare [104]	P497H, P506S, T487T, T487I, et al	autophagy, mitophagy, and neuroinflammation by protein blocked protein degradation [105]	Mainly presence with FTD	0.98 (0.40, 2.5)
*CCNF*	Cyclin F	16p13.3	Strong evidence	rare [106]	A74T, E528Q, E624K et al	neurotoxicity and motor neuron degeneration by defective protein degradation systems and the pathological accumulation of a protein involved in RNA processing and metabolism [107]	NA	2.9 (0.19, 45.0)

*ALSoD* Amyotrophic Lateral Sclerosis online Database [4], *NA* not Available

^a^Total HR (95%CI) were calculated in this study

The rankogram plots are shown in Fig. 3. According to the surface under the cumulative ranking curve (SUCRA), *ATXN2* polyQ repeats (≥31) had the largest probability of being the worst genetic variants (SUCRA = 86.3%, data not shown). *TBK1*(70.0%), *FUS* (69.5%), *CCNF* (67.7%), and *C9orf72* (63.4%) had a similar probability of being the worst. The result of pairwise comparison among these genes is shown in Fig. 3.

Furthermore, there is no significant difference between direct and indirect meta-analysis (Additional file 1: Fig. S1) and the heterogeneity between studies is also acceptable (Additional file 1: Fig. S2-S12), indicating the results of NMA are reliable. As for publication bias, we did not find significant funnel plot asymmetry in studies reporting *C9orf72* expansion in patients with ALS compared to those without ALS-related mutation (Additional file 1: Fig. S13).

### Four modification loci in ALS-related genes by pairwise meta-analysis

**Fig. 4:**
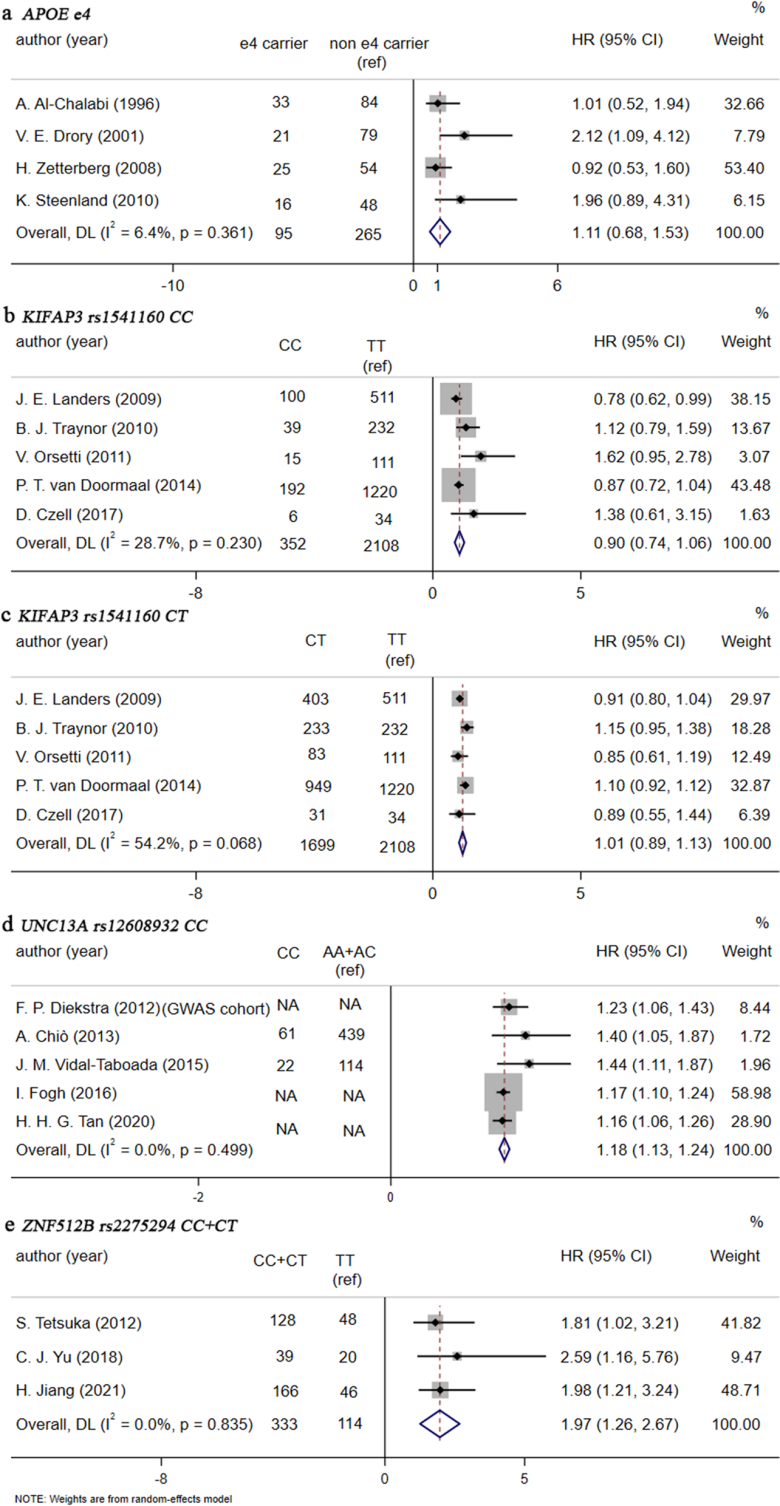
Forest plots of HR for modification loci included in pairwise meta-analysis. **a** Forest plot of HR for *APOE* ε4. **b** Forest plot of HR for *KIFAP3* rs1541160 CC. **c** Forest plot of HR for *KIFAP3* rs1541160 CT. **d** Forest plot of HR for *UNC13A* rs12608932 CC. **e** Forest plot of HR for *ZNF512B* rs2275294 CC+TT. HR, hazard ratio; CI, confidence interval

Four modification loci in ALS-related genes, *APOE* ε4 allele, *KIFAP3* rs1541160, *UNC13A* rs12608932, and *ZNF512B* rs2275294, were available for pairwise meta-analysis (Additional file 1: Table S2). In this study, *UNC13A* rs12608932 CC (recessive model) and *ZNF512B* rs2275294 CC+CT (dominant model) could accelerate the death of ALS patients (HR 1.18 and 1.97, respectively, Fig. 4). However, *APOE* ε4 allele, *KIFAP3* rs1541160 CC or CT (additive model) did not show any modificatory effect on ALS survival (Fig. 4). And no obvious heterogeneity was found.

### Other loci in ALS-related genes by systematic review

The other loci in ALS-related genes, which were not suitable for pool analysis, were reviewed systematically and shown in Additional file 1: Table S3. We found the minor allele carriers of *CX3CR1* V249I, *KCNJ11* rs5219, *LXRs* rs2695121, *PRGN* rs34424835, *HTR2B* rs10199752, *PON1* rs662, *SLC11A2* rs407135, *CAMTA1* s2412208, and *IDE* rs139550538 might have shorter survival than that without minor allele carriers, but *ABCC8* rs4148646 GG, *KCNJ11* rs5219 TT, *HTR2B* rs10199752 AA, and *C7* gene rs3792646 AA might be related to more prolonged survival. Additionally, *SMN1* and *SMN2* copy numbers, and the remaining genes on display did not show any modificatory effect on ALS survival. However, there were not enough articles for meta-analysis.

## Discussion

Genetic factors play a pivotal role in the pathogenesis and phenotypic modification of ALS. How the genetic factors affected the survival of ALS was largely unknown yet, especially for variants in the ALS causative genes, in consideration of the limited sample with genetic mutation. This study is the first systematic analysis for the effect of comprehensive genetic factors on the survival of ALS. Using NMA and pairwise meta-analysis, we found three genes, including *ATXN2* polyQ, *C9orf72* repeats, and *FUS* variants, and two genetic modifiers, *ZNF521B* rs2275294 C allele and *UNC13A* rs12608932 CC genotype, were associated with short survival of ALS.

This current study provided robust evidence that genetic factors affected the survival of ALS. For NMA, among the nine ALS causative/risk genes involved, we found *C9orf72*, *ATXN2*, and *FUS* were associated with much shorter survival. Ataxin 2 is a protein-coding gene and the N-terminal region of this protein contains a polyglutamine tract of 14-31 residues that can be expanded in the pathogenic state to 32-200 residues. The long trinucleotide repeats expansions (≥36) in *ATXN2* have been known to be related to spinocerebellar ataxia type 2 (SCA2) [108], but the intermediate length expansions (27–33 repeats) were also discovered to increase susceptibility to ALS [88], not only in FALS but also in SALS [109]. Meanwhile, another study also reported that *ATXN2* ployQ may render *C9orf72* repeat expansion carriers more susceptible to ALS [110]. It was also associated with a more severe phenotype and a worse prognosis of ALS, causing a significantly shorter survival (1.2 vs. 4.2 years) [26]. In this study, we found it had the most significant probability of becoming the genetic background with the worst prognosis for ALS (HR=3.6). Ataxin-2 plays a critical role in the normal physiological functions of cells, including RNA processing, receptor endocytosis, the formation of stress granules, and induction of aberrant TDP-43 cleavage [93]. In ALS, it altered protein homeostasis and RNA metabolism, leading to neurotoxicity [1, 94]. Motor neurons in this condition may degenerate faster than those without *ATXN2* ployQ expansion or with other genetic variants. Therefore, several gene therapies targeted for *ATXN2* used in cell or ALS animal models to prevent or delay its progression were reported [111].

Thanks to advances in the next-generation technology, *C9orf72* GGGGCC (G_4_C_2_) hexanucleotide repeat expansion was identified as the most common mutation in Europe ALS and the second most common mutation in Asia ALS [95]. Hence, whether the clinical studies are based on genetics and biomarkers, or the basic research based on pathogenies mechanisms or therapeutics, *C9orf72* was the most studied gene in the ALS research field in the last ten years. Although healthy individuals can have from 2 to 25 G_4_C_2_ repeats, ALS or FTD patients harbor hundreds to even thousands of these repeats [112, 113]. Furthermore, it is involved with RNA foci-mediated toxicity, dipeptide repeat protein (DPR)-mediated toxicity and/or loss-of-function due to reduced levels of C9orf72 protein [96]. Here, after synthesizing a total of 40 comparisons, we found *C9orf72* expansion was also associated with shorter survival (HR:1.6, 95%CI [1.4, 1.9]). In addition, cases with *C9orf72* expansion may have a more rapid rate of cognitive decline and a higher risk of developing FTD [114], which were predictors for poor prognosis for ALS as well [5]. So far, gene therapy targeting *C9orf72* has been carried out in preclinical studies [115, 116], and these methods are promising approaches for future in vivo studies.

*SOD1*, *FUS*, and *TARDBP*, the other three common ALS causative genes, usually present as missense variants. When all the reported mutation sites were analyzed together, we found only *FUS* would shorten the survival time of ALS. However, we could not ignore that different variants on these genes have different effects on ALS survival. Based on previous genetic studies, we summarized the characteristics of several common variants in these genes in Additional file 1: Table S5 [117–119]. The most significant heterogeneity may exist in *SOD1* with more than 150 ALS-associated *SOD1* variants described*.* SOD1 was believed to cause ALS through toxic gain of function caused by aggregation of misfolded SOD1 [1, 97]. Variants with different influences on survival are usually located in different domains and are more likely to have a strikingly different effect on protein structure and function. For example, *SOD1*^A4V^ and *SOD1*^H46R^ associated, with the shorter and significantly longer durations, respectively, were identified not only in Caucasians but also in Asians. Our recent cohort study has yielded similar results [13]. Thus, the results obtained in the current study may be due to a mixture of different variants in *SOD1*. There, it does not mean that *SOD1* variants do not affect the survival of ALS, for individuals, specific variants should be identified. Further, we found a similar but slight difference from a meta-analysis of published *FUS*-ALS cases [119]. It was found to have a different duration from onset to the severe event among *FUS*^R521^, *FUS*^P525L^, frameshift/truncation, and the remaining variants in *FUS* [119]. However, there did not seem to be a mutation in *FUS* reported that would prolong the survival of ALS (Additional file 1: Table S5) [11–13, 119]. Taken together, it appears that genetic testing of *FUS* is indicated in patients with shorter disease duration. *TARDBP* gene encoded TDP-43, a DNA-/RNA-binding protein normally localized to the nucleus [120], which is also the most widespread and pathologic hallmark in the ALS/FTD spectrum [94]. Although *TARDBP* did not get a definitive positive result in this study, it seems tempting to suggest that TARDBP-ALS cases might have a better prognosis because the HR was 0.77 and 95% CI was 0.61 to 1. Moreover, there was no shortage of reports of *TARDBP*-ALS cases that have survived for decades [12]. Similarly, there are differences in the impact of different variants on the survival of ALS.

However. we did not find *TBK1*, *NEK1*, *UBQLN2*, and *CCNF* were associated with survival of ALS either. Maybe there were too few studies or limited patients with variants in these genes to reach the significant difference. The features for these genes were also shown in Table 1. It must be emphasized that those genes included in NMA have been regarded as ALS risk genes with strong evidence or above, but some of the variants in them might not be of demonstrated pathogenetic significance. For instance, most pathogenic variants identified in *TBK1* are concentrated within the kinase and the coiled-coiled domains [121], which usually were loss-of-function types, while for some missense variants (such as p.K291E, p.I305T, p.L306I, p.H322Y, p.T3221I, p.R444Q, and p.A535T) may need more functional studies to research their pathogenicity [122]. And *NEK1* missense variants confer risk of developing ALS is also a matter of debate [123], the similar condition exists in other genes as well. While repeat expansions in genes are more susceptible to reaching the statistical significance threshold. This limit due to our methods may have an impact on the results.

In addition, four genetic factors were studied by pairwise meta-analysis. Consistent with most previous studies [6, 8, 59, 60, 124], *UNC13A* rs12608932 CC was a predictor of poor survival in ALS. The rs12608932 in *UNC13A* is associated with ALS/FTD susceptibility [125] and may indicate poorer cognitive functioning, higher rates of behavioral impairment, and higher rates of FTD [60]. A previous meta-analysis for a single factor has already shown this effect [126]. Therefore, this genotype may function as a prognostic indicator and could be used to define patient endophenotypes in clinical trials. Although the mechanisms by which the CC genotype of rs12608932 in *UNC13A* significantly decreased the survival time of ALS patients was not entirely clear, the very recent studies found *UNC13A* variants exacerbate the effects of reduced TDP-43 function and TDP-43 can repress a cryptic exon-splicing event in *UNC13A* [127, 128]. Hence, it may provide a promising therapeutic target for TDP-43 proteopathies. We also detected that the *ZNF521B* rs2275294 C allele indicates a poor prognosis of ALS, meanwhile a meta-analysis suggested that the *ZNF512B* rs2275294 polymorphism is also associated with ALS risk [129]. The *ZNF512B* gene encodes a protein of 893 amino acids, which is expressed in the brain and spinal cord. Upregulation of ZNF512B activates TGF-β/Smad signaling, while downregulation inhibits this pathway and rs2275294C may reduce this neuroprotective TGF-β/Smad signaling [130]. Several other genes (such as *EPHA4*, *CAMTA1*, and *HFE*) may be associated with the prognosis of ALS, however, they cannot be performed by meta-analysis due to the limited studies.

It is controversial whether *APOE* polymorphism can change the course of ALS [56, 57]. However, *APOE* ε4 allele does not modify the clinical course of ALS as well under meta-analysis, so does *KIFAP3* rs1541160. As for the remaining genetic factors, although *SMN* genes have been reported to be associated with ALS survival [69, 131], This conclusion was not supported based on the results of a recent and large study including new and all previously reported results on *SMN1* and *SMN2* copy number variation in ALS [87]. .The genetic mechanisms of *SMN1* and *SMN2* are implicated in motor neuron death in spinal muscular atrophy, but SMN expression levels in the physiological range may not modify the progression of ALS [87]. Additionally, a very recent large GWAS also looked at the role of some genetic factors mentioned above on modifying ALS survival and found the effect of common genetic risk factors for ALS susceptibility on disease progression was limited [132], suggesting the influence of a single SNP or gene on ALS survival cannot be magnified.

Although we find some potential genetic factors that affected the survival of ALS, these findings should be interpreted with caution, given some limitations. First and foremost, different variants in the same gene might have different effects on survival, it may result in information missing when a gene was analyzed as a whole factor. What is more, as we discussed above, whether some of the variants in these genes are ALS-associated mutations remains in doubt. Therefore, the results were not absolutely correct. Besides, some studies did not report the HR directly, when we get HR from the survival curve, there may be some slight gap with the accurate results. Some differences in follow-up time, the included population, and the definition of outcomes may lead to publication bias and the non-genetic factors related to ALS survival may also play a role [5]. Moreover, the lack of commonality of prognostic factors investigated in different cox PH models is also a limitation, and some studies are single-factor analyses and did not adjust for confounding factors. In addition, the small number of prospective studies was also a limitation. For example, *ZNF521B* rs2275294 were reported in only three articles, the results should be explained with caution. Therefore, more high-quality prospective studies are warranted. Finally, the rules of the review methodology of its restriction to articles published in English, and the low specificity of the search strategy, increase the risk of missing relevant studies.

## Conclusions

The present meta-analysis summarized and contrasted evidence for genetic prognostic factors in patients with ALS and will help to understand ALS genetics. Genetic prognostic factors deserve attention and careful consideration as the field moves forward to combat and prevent this devastating disease.

## Supplementary Information

**Additional file 1: Table S1.** Characteristics of the articles included in network meta-analysis for variants in ALS causative genes. **Table S2.** Characteristics of the articles included in pairwise meta-analysis for other modification loci. **Table S3.** Characteristics of the articles included in systematic review for other modification loci. **Table S4.** The quality of articles included in network meta-analysis and pairwise meta-analysis by NOS. **Table S5.** The feathers for different variants in SOD1, FUS, TARDBP. **Figure S1.** Forest plot for inconsistency test in network meta-analysis. **Figure S2-S12.** Heterogeneity analysis n network meta-analysis. **Figure S13.** publication bias for studies reporting C9orf72 expansion in patients with ALS compared to those without ALS-related mutation.

## Data Availability

All data generated or analyzed during this study are included in this published article and its supplementary information files. Effect size and study details were extracted from the original papers, which are available in the public domain.

## Abbreviations

ALS: Amyotrophic lateral sclerosis
CI: Confidential intervals
DPR: Dipeptide repeat protein
FALS: Family amyotrophic lateral sclerosis
FTD: Frontotemporal dementia
GWAS: Genome-Wide Association Study
HR: Hazard ratio
ISPOR: International Society for Pharmacoepidemiology and Outcomes Research
NIV: Noninvasive ventilation
NMA: Network meta-analysis
NOS: Newcastle-Ottawa Scale
PH: Proportional hazard
PRISMA: Preferred Reporting Items for Systematic Reviews and Meta-Analyses
RCT: Randomized clinical trial
SALS: Sporadic amyotrophic lateral sclerosis
SCA2: Spinocerebellar ataxia type 2
SNP: Single nucleotide polymorphism
SUCRA: Surface under the cumulative ranking curve
