# Supplementary material for: Genetic factors for survival in amyotrophic lateral sclerosis: an integrated approach combining a systematic review, pairwise and network meta-analysis
Source: BMC Med. 2022 Jun 27;20:209. doi: 10.1186/s12916-022-02411-3 (PMC9235235; doi:10.1186/s12916-022-02411-3)
Supplement: Supplementary file 1 — Additional file 1: Table S1. Characteristics of the articles included in network meta-analysis for variants in ALS causative genes. Table S2. Characteristics of the articles included in pairwise meta-analysis for other modification loci. Table S3. Characteristics of the articles included in systematic review for other modification loci. Table S4. The quality of articles included in network meta-analysis and pairwise meta-analysis by NOS. Table S5. The feathers for different variants in SOD1, FUS, TARDBP. Figure S1. Forest plot for inconsistency test in network meta-analysis. Figure S2-S12. Heterogeneity analysis n network meta-analysis. Figure S13. publication bias for studies reporting C9orf72 expansion in patients with ALS compared to those without ALS-related mutation. [file 12916_2022_2411_MOESM1_ESM.docx]

**Genetic predictors for survival in amyotrophic lateral sclerosis: a systematic review,** **pairwise and network meta-analysis**

**SUPPLEMENTARY MATERIALS**

| **Section** | **Description** | **Pages** |
| --- | --- | --- |
| **Supplementary Tables** |  |  |
|  | **Table S1** Characteristics of the articles included in network meta-analysis for variants in ALS causative genes. | 2-8 |
|  | **Table S2** Characteristics of the articles included in pairwise meta-analysis for other modification loci. | 9-12 |
|  | **Table S3** Characteristics of the articles included in systematic review for other modification loci. | 13-19 |
|  | **Table S4** The quality of articles included in network meta-analysis and pairwise meta-analysis by NOS. | 20-21 |
|  | **Table S5** The feathers for different variants in *SOD1*, *FUS*, *TARDBP*. | 22 |
| **Supplementary Figures** |  |  |
|  | **Figure S1** Forest plot for inconsistency test in network meta-analysis | 23 |
|  | **Figure S2-S12** Heterogeneity analysis n network meta-analysis. | 24-34 |
|  | **Figure S13** publication bias for studies reporting *C9orf72* expansion in patients with ALS compared to those without ALS-related mutation | 35 |

**Table S1 Characteristics of the articles included in network meta-analysis for variants in ALS causative genes.**

**Table S1-1 articles with 2-arm studies**

| **2-arm studies: *ATXN2* polyQ repeats (****≥ 31) vs without *ATXN2* polyQ repeats (<31)** | | | | | | | | | | | |
| --- | --- | --- | --- | --- | --- | --- | --- | --- | --- | --- | --- |
| First author | Year | Country/region | Type of study | Number of ALS patients | Number of patients with variants | Median or mean survival from onset | | Diagnosis | | HR (95%CI) | HR source* |
|  |  |  |  |  |  | patients with variants | patients without variants | Diagnostic criteria | level |  |  |
| G. Borghero | 2015 | Sardinian | retrospective cohort | 375 | 4 | median 1.2 y, IQR (0.9-2.7) | median 4.2y, IQR (2.2-10） | revised El Escorial criteria | definite, probable, probable  laboratory-supported or possible ALS | 4.71 (1.75, 12.67) | Estimated |
| A. Chiò (discovery cohort) | 2015 | Italy | prospective cohort | 672 | 19 | median 1.8y, IQR (1.3-2.2) | median 2.7y, IQR (1.6-5.1) | revised El Escorial criteria | definite, probable, and probable  laboratory-supported ALS | 2.79 (1.67, 4.64) | Available |
| A. Chiò (validation cohort) | 2015 | Rome, Italy | prospective cohort | 661 | 16 | median 2.0y, IQR (1.3-5.4) | median 3.2y, IQR (2.0-6.4) | revised El Escorial criteria | definite, probable, and probable  laboratory-supported ALS | 5.38 (3.71, 7.81) | Estimated |
| **2-arm studies: *C9orf72* repeat expansion (>30) vs without *C9orf72* repeat expansion (≤30)** | | | | | | | | | | | |
| S. Byrne | 2012 | Ireland | retrospective cohort | 191 | 21 | median 1.7y, 95%CI (0.74–2.60) | median 2.2y, 95%CI (1.76–2.56) | NA | NA | 1.9 (1.1, 3.7) | Available |
| A. Ratti | 2012 | Italy | retrospective case-control | 1534 | 128 | median 1.3y | median 2.3y | revised El Escorial criteria | NA | 2.61 (2.21, 3.1) | Estimated |
| M. Sabatelli | 2012 | Italy | retrospective cohort | 1757 | 69 | median 2.7y, 95%CI (2.1–3.3) | median 3.6y, 95% CI (3.3–3.8) | revised El Escorial criteria | definite, probable, probable laboratory-supported or possible ALS | 1.79 (1.26, 2.98) | Available |
| W. van Rheenen | 2012 | Netherlands | prospective cohort | 1759 | 171 | median 2.5y, range (0.45–13.0) | median 2.7y, range (0.13–31.8) | revised El Escorial criteria | possible, probable laboratory-supported, probable, or definite ALS | 1.46 (1.17, 1.83) | Available |
| S. Millecamps | 2012 | France | retrospective cohort | 170 | 130 | median 2.42y, range (0.25y-7.08y) | meidan 3.25y, range (0.67-28) | NA | probable or definite ALS | 1.9 (1.33, 2.7) | Estimated |
| S. Debray(sALS cohort) | 2013 | Belgium | retrospective cohort | 62 | 32 | mean±SD (2.45±1.58) | mean±SD (5.64y±4.97) | revised El Escorial or Awaji criteria | probable ALS | 2.54 (1.52,4.27) | Available |
| S. Debray(fALS cohort) | 2013 | Belgium | retrospective cohort | 471 | 45 | mean±SD (2.55±1.51) | mean±SD (3.03y±1.96) | revised El Escorial or Awaji criteria | probable ALS | 1.09 (0.78,1.52) | Available |
| A. García-Redondo | 2012 | Spain | retrospective cohort | 936 | 67 | median 3.42y, range(2.83,3.92) | median 4.42y, range(3.75,5.0) | revised El Escorial criteria | definite or probable ALS | 1.93 (1.51,2.48) | Estimated |
| D. J. Irwin | 2013 | USA | retrospective case-control | 67 | 31 | mean±SD (2.6y±0.3) | mean±SD (3.8y±0.4) | revised El Escorial criteria | NA | 1.91 (1.1,3.31) | Estimated |
| K. Van Laere | 2014 | Leuven, Belgium | prospective cohort | 70 | 11 | NA | NA | revised El Escorial and Awaji-Shima criteria | NA | 1.43 (0.39, 5.21) | Estimated |
| A. Calvo | 2016 | Italy | prospective cohort | 526 | 33 | NA | NA | revised El Escorial criteria | possible, probable laboratory supported, probable, definite ALS | 1.66 (1.16, 2.39) | Available |
| R. Govaarts | 2016 | Netherlands | prospective cohort | 110 | 3 | NA | NA | revised El Escorial criteria | possible, probable laboratory supported, probable, definite ALS | 1.54 (1.17,2.03) | Available |
| M. E. Umoh | 2016 | USA | retrospective cohort | 781 | 61 | median 2.4y, 95%CI (1.9–2.9) | median 4.3y, 95%CI (3.8–4.7) | revised El Escorial criteria | NA | 2.16 (1.596,2.927) | Available |
| T. F. Gendron | 2017 | USA | prospective cohort | 106 | 68 | NA | NA | revised El Escorial criteri | NA | 4.08 (2.24,7.43) | Available |
| W. Reniers | 2017 | Belgium | prospective cohort | 364 | 56 | NA | NA | revised El Escorial or Awaji criteria | at least possible ALS | 2.52 (1.37, 4.64) | Available |
| G. Miltenberger-Miltenyi | 2019 | Portugal | prospective cohort | 344 | 31 | NA | NA | revised El Escorial criteria | NA | 2.05 (1.46,2.89) | Estimated |
| A. J. Cammack | 2019 | USA | retrospectiv  cohort | 413 | 199 | median 2.49y | median 2.50y | revised El Escorial criteria | a minimum diagnosis of possible ALS | 1.2 (0.99,1.45) | Estimated |
| J. Rooney | 2019 | Ireland | retrospective cohort | 630 | 58 | NA | NA | El Escorial criteria | NA | 1.62 (1.21, 2.15) | Available |
| F. Trojsi | 2019 | Italy | retrospective cohort | 728 | 47 | median 2.95y, 95%CI (2.15–3.67) | median 3.67y, 95%CI (3.33–4.0) | revised El Escorial criteria | definite or clinical and laboratory-supported probable ALS | 1.58 (1.07, 2.33) | Available |
| M. Benatar | 2020 | USA | prospective cohort | 229 | NA | NA | NA | NA | NA | 1.7 (0.91,3.18) | Available |
| M. De Schaepdryver | 2020 | Belgium, Italy | retrospective cohort | 269 | NA | NA | NA | revised El Escorial criteria | suspected, possible, probable and definite ALS | 1.08 (0.62,1.89) | Available |
| D. Brand | 2021 | USA | retrospective cohort | 1298 | 91 | NA | NA | El Escorial criteria | clinically definite, probable, possible, or suspected ALS | 2.06 (1.61,2.65) | Available |
| U. Klappe | 2021 | Sweden | retrospective case-control | 93 | 10 | NA | NA | revised El Escorial criteria | clinically definite, probable, and possible ALS | 0.68 (0.14,3.2) | Available |
| F. Puentes | 2021 | UK | prospective cohort | 37 | 11 | NA | NA | revised El Escorial criteria | NA | 2.39 (1.4,4.12) | Estimated |
| **2-arm studies: *SOD1* vs none** | | | | | | | | | | | |
| M. E. Cudkowicz | 1997 | USA and Europe | retrospective case-control | 318 | 184 | median 2.1y | median 2.0y | revised El Escorial criteria | NA | 0.9 (0.71,1.14) | Estimated |
| **2-arm studies: *FUS* vs none** | | | | | | | | | | | |
| A. Hubers | 2015 | Germany | prospective cohort | 14 | 6 | median 3.33, 95%CI (0.58-3.33) | median NA, 95%CI (3.5, NA) | revised El Escorial criteria | NA | 6.89 (1.15,41.4) | Estimated |

**Table S1-2 articles with 3-arm studies**

| **3-arm studies: *TARDBP* vs *SOD1* vs none** | | | | | | | | | | | | | | | |
| --- | --- | --- | --- | --- | --- | --- | --- | --- | --- | --- | --- | --- | --- | --- | --- |
| First author | Year | Country/region | Type of study | Number of ALS patients | Number of patients with mutation | | | | Median or mean survival from onset | | | Diagnosis | | HR (95%CI) | HR source |
|  |  |  |  |  | *TARDBP* | | *SOD1* | |  |  |  |  |  |  |  |
|  |  |  |  |  |  |  |  |  | *TARDBP* | *SOD1* | none(ref) | Diagnostic criteria | level |  |  |
| P. Corcia | 2012 | France | retrospective cohort | 823 | 28 | | 58 | | median 5.25y, 95%CI (2.67–6.43) | median 3.42y, 95%CI (2.08–4.10) | median 2.56y, 95%CI (2.42–2.67) | revised El Escoril criteria | NA | *TARDBP*:0.59 (0.36,0.95)  *SOD1*: 0.78 (0.57,1.06) | Estimated |
| **3-arm studies: *TARDBP* vs *FUS* vs *SOD1*** | | | | | | | | | | | | | | | |
| First author | Year | Country/region | Type of study | Number of ALS patients | Number of patients with mutation | | | | Median or mean survival from onset | | | Diagnosis | | HR (95%CI) | HR source |
|  |  |  |  |  | *TARDBP* | *FUS* | | *SOD1* |  |  |  |  |  |  |  |
|  |  |  |  |  |  |  |  |  | *TARDBP* | *FUS* | *SOD1(ref)* | Diagnostic criteria | level |  |  |
| W. Liu | 2021 | China | retrospective cohort | 242 | 53 | 28 | | 159 | median 9.67y, range (0.82–18) | median 3.0y, range (1.0–6.67y) | median5.0y, range (0.5–20) | revised El Escoril criteria | definite, probable, probable laboratory-supported ALS | *TARDBP*: 0.81(0.56,1.18)  *FUS*: 2.12(1.4,3.21) | Estimated |

**Table S1-3 articles with 4-arm studies**

| **4-arm studies:*C9orf72* vs *TARDBP* vs *SOD1* vs none** | | | | | | | | | | | | | | | |
| --- | --- | --- | --- | --- | --- | --- | --- | --- | --- | --- | --- | --- | --- | --- | --- |
| First author | Year | Country/region | Type of study | Number of ALS patients | Number of patients with mutation | | | Median or mean survival from onset | | | | Diagnosis | | HR (95%CI) | HR source |
|  |  |  |  |  | *TARDBP* | *C9orf72* | *SOD1* |  |  |  |  |  |  |  |  |
|  |  |  |  |  |  |  |  | *TARDBP* | *C9orf72* | *SOD1* | none(ref) | criteria | level |  |  |
| S. Lattante | 2012 | Lazio, Italy | retrospective cohort | 462 | 13 | 12 | 10 | median 5.50y | median 3.12y | median 8.42y | median 3.30y | El Escoril criteria | definite, probable, or laboratory-supported probable ALS | *TARDBP*: 0.89(0.49,1.62)  *C9orf72*: 3.07(1.85,5.01)  *SOD1*: 0.47(0.21,1.04) | Estimated |
| G. Borghero | 2014 | Sardinia, Italy | retrospective cohort | 350 | 75 | 51 | 4 | median 6.5y, IQR (3.3±10.5) | median 2.7y, IQR(1.9±3.8) | NA | NA | revised El Escoril criteria | definite, probable, probable laboratory-supported, possible ALS | *TARDBP*: 0.83(0.6,1.16)  C9orf72: 2.24(1.63,3.07)  *SOD1*: 0.71(0.18,2.84) | Estimated |
| **4-arm studies: *TARDBP* vs *FUS* vs *SOD1* vs none** | | | | | | | | | | | | | | | |
| First author | Year | Country/region | Type of study | Number of ALS patients | Number of patients with mutation | | | Median or mean survival from onset | | | | Diagnosis | | HR (95%CI) | HR source |
|  |  |  |  |  | *TARDBP* | *FUS* | *SOD1* |  |  |  |  |  |  |  |  |
|  |  |  |  |  |  |  |  | *TARDBP* | *FUS* | *SOD1* | none(ref) | criteria | level |  |  |
| S. Millecamps | 2010 | France | retrospective cohort | 233 | 23 | 29 | 55 | median 4.25y, SE 0.75 | median  2.17y, SE 0.25 | median 3.0y, SE 0.83 | median 2.67y,  SE 0.17 | El Escoril criteria | probable or definite ALS | *TARDBP*: 0.71(0.46,1.1)  *FUS*: 1.48(1.01,2.17)  *SOD1*: 0.64(0.47,0.88) | Estimated |

**Table S1-4 articles with 5-arm studies**

| **5-arm studies: *C9orf72* vs *TARDBP* vs *FUS* vs *SOD1* vs none** | | | | | | | | | | | | | | | | | |  |
| --- | --- | --- | --- | --- | --- | --- | --- | --- | --- | --- | --- | --- | --- | --- | --- | --- | --- | --- |
| First author | Year | Country/region | Type of study | Number of ALS patients | Number of patients with mutation | | | | Median or mean survival from onset | | | | | Diagnosis | | HR (95%CI) | HR source |  |
|  |  |  |  |  | *C9of72* | *TARDBP* | *FUS* | *SOD1* |  |  |  |  |  |  |  |  |  |  |
|  |  |  |  |  |  |  |  |  | *C9of72* | *TARDBP* | *FUS* | *SOD1* | none(ref) | criteria | level |  |  |  |
| A. Chiò | 2012 | Italy | prospective cohort | 177 | 45 | 13 | 6 | 38 | median 3.2y, 95%CI (2.9–3.4) | median 5.0y, 95% CI (3.6–7.2) | median 1.9y, 95% CI (1.7–2.1) | median 3.8y, 95% CI (3.1–6.5) | NA | NA | NA | *C9orf72*: 2.44 (1.55,3.84)  *TARDBP*: 1.82 (0.66,5.05)  *FUS*: 6.29 (2.44,16.22)  *SOD1*: 1.66 (1.01,2.71) | Estimated |  |

**Table S1-5 articles with 6-arm studies**

| **6-arm studies: *C9orf72* vs *TARDBP* vs *FUS* vs *UBQLN2* vs *CCNF* vs *SOD1*** | | | | | | | | | | | | | | | | | | | | |  |
| --- | --- | --- | --- | --- | --- | --- | --- | --- | --- | --- | --- | --- | --- | --- | --- | --- | --- | --- | --- | --- | --- |
| First author | Year | Country/region | Type of study | Number of ALS patients | Number of patients with mutation | | | | | | Median or mean survival from onset | | | | | | Diagnosis | | HR (95%CI) |  |  |
|  |  |  |  |  | *C9orf72* | *TARDBP* | *FUS* | *UBQLN2* | *CCNF* | *SOD1* |  |  |  |  |  |  |  |  |  | HR source |  |
|  |  |  |  |  |  |  |  |  |  |  | *C9orf72* | *TARDBP* | *FUS* | *UBQLN2* | *CCNF* | *SOD1(ref)* | criteria | level |  |  |  |
| E. P. McCann | 2017 | Australia | retrospective cohort | 265 | 121 | 5 | 27 | 8 | 7 | 97 | NA | NA | NA | NA | NA | NA | El Escorial criteria | definite, probable, probable laboratory-supported ALS | *C9orf72*: 1.39 (1.03,1.88)  *TARDBP*: 1.01 (0.39,2.61)  *FUS*: 2.06 (1.29,3.3)  *UBQLN2*: 1.07 (0.53,2.18)  *CCNF*: 3.05 (1.35,6.85) | Estimated |  |

**Table S1-6 articles with 7-arm studies**

| **7-arm studies: *C9orf72* vs *SOD1*vs *TARDBP* vs *FUS* vs *TBK1*vs *NEK1* vs none** | | | | | | | | | | | | | | | | | | | | | |
| --- | --- | --- | --- | --- | --- | --- | --- | --- | --- | --- | --- | --- | --- | --- | --- | --- | --- | --- | --- | --- | --- |
| First author | Year | Country/region | Type of study | Number of ALS patients | Number of patients with mutation | | | | | | Median or mean survival from onset | | | | | | | Diagnosis | | HR (95%CI) | HR source |
|  |  |  |  |  | *C9orf72* | *SOD1* | *TARDBP* | *FUS* | *TBK1* | *NEK1* |  |  |  |  |  |  |  |  |  |  |  |
|  |  |  |  |  |  |  |  |  |  |  | *C9orf72* | *SOD1* | *TARDBP* | *FUS* | *TBK1* | *NEK1* | none (ref) | criteria | level |  |  |
| Y. P. Chen | 2021 | China | retrospective cohort | 1496 | 23 | 43 | 15 | 19 | 10 | 10 | median 2.58y, 95% CI (2.12 -3.04) | median 2.54y, 95% CI (2.09-2.99) | median 2.98y, 95% CI (2.63-3.33) | median 2.98y, 95% CI (2.62-3.33) | median 2.24y, 95% CI (1.40-3.08) | median 5.04y, 95% CI (2.08-7.99) | median 3.55y, 95% CI (3.33- 3.78) | revised El Escoril criteria | NA | *C9orf72*: 2.81(1.81,4.36)  *SOD1*: 1.84(1.33,2.56)  *TARDBP*: 3.93(2.39,6.47)  *FUS*: 2.35(1.55,3.58)  *TBK1*: 2.88(1.61,5.15)  *NEK1*: 1.44(0.85,2.43) | Estimated |

*‘Estimated’: HR was evaluated from Kaplan-Meier curve or other curves by Engauge Digitizer version 12.1, and HRs and 95 % CI were estimated by using Richard Steven’s excel workbook. ‘Available’: HR was reported in the article. ALS: amyotrophic lateral sclerosis; HR: hazard ratio; CI: confidence interval; SD: standard deviation; NA: not available. IQR: interquartile range.

**Table S2 Characteristics of the articles included in pairwise meta-analysis for other modification loci.**

**Table S2-1 *APOE*** ε4

| ***APOE* ε4 vs non-ε4 carriers** | | | | | | | | | | | | | |
| --- | --- | --- | --- | --- | --- | --- | --- | --- | --- | --- | --- | --- | --- |
| First author | Year | Country/region | Type of study | Total ALS patients | Number of ε4 carriers | Number of non-e4 carriers | Median or mean survival from onset | | Diagnosis | | HR (95%CI) | HR source | systematic review or pairwise meta-analysis |
|  |  |  |  |  |  |  | ε4 carriers | non-ε4 carriers(ref) | criteria | level |  |  |  |
| A. Al-Chalabi | 1996 | England | prospective cohort | 117 | 33 | 84 | median 2.5y, 95%CI (1.67-4.08) | median 4.08y, 95%CI (3.0-3.5) | NA | NA | 1.01 (0.52,1.94) | Available | meta-analysis |
| V. E. Drory | 2001 | Israel | retrospective cohort | 100 | 21 | 79 | median 3.17y | median 3.0y | revised El Escorial criteria | definite or probable ALS | 2.12 (1.09,4.12) | Estimated | meta-analysis |
| H. Zetterberg | 2008 | Sweden | retrospective cohort | 79 | 25 | 54 | For one ε4-carriers: median5.6y, range (2.0–16) in sALS; median2.6y, range (0.75–4.0) in fALS; For two ε4-carriers: median 3.5y, range (1.4–9.4) in sALS; median 5.1y in fALS; | median 3.8y, range (2.3–26) in sALS; median 2.1y, range (0.67–14) in fALS | revised El Escorial criteria | NA | 0.92 (0.53,1.6) | Estimated | meta-analysis |
| K. Steenland | 2010 | USA | retrospective cohort | 64 | 16 | 48 | NA | NA | NA | NA | 1.96(0.89,4.31) | Available | meta-analysis |

**Table S2-2 *UNC13A* rs12608932**

| ***UNC13A* rs12608932 CC vs AC + CC** | | | | | | | | | | | | | |
| --- | --- | --- | --- | --- | --- | --- | --- | --- | --- | --- | --- | --- | --- |
| First author | Year | Country/region | Type of study | Total ALS patients | Number of CC carriers | Number of AC or CC carriers | Median or mean survival from onset | | Diagnosis | | HR (95%CI) | HR source | systematic review or pairwise meta-analysis |
|  |  |  |  |  |  |  | CC carriers | AC or CC carriers(ref) | criteria | level |  |  |  |
| F. P. Diekstra  (GWAS cohort) | 2012 | Netherlands, Belgium, Sweden | retrospective case-control | NA | NA | NA | NA | NA | El Escorial criteria | possible, probable, or definite ALS | 1.23(1.06,1.43) | Available | meta-analysis |
| A. Chiò | 2013 | Italy | retrospective case-control | 500 | 61 | 439 | median 2.5 y, IQR, (1.6, 4.2) | median3.5 y, IQR, (2.2, 6.4) | revised El Escorial criteria | probable, probable laboratory-supported, or definite ALS | 1.4(1.05,1.87) | Available | meta-analysis |
| J. M. Vidal-Taboada | 2015 | Spain | retrospective case-control | 136 | 22 | 114 | median 2.0 y, 95%CI (1.78, 2.22) | median4.75 y, 95%CI (4.08, 5.43) | revised El Escorial criteria | Definite ALS, Probable ALS, or Probable ALS-Laboratory supported | 1.439(1.105,1.873) | Available | meta-analysis |
| I. Fogh | 2016 | Europe, USA | retrospective case-control | 3574 | NA | NA | NA | NA | revised El Escorial criteria | NA | 1.17 (1.1,1.24) | Available | meta-analysis |
| H. H. G. Tan | 2020 | Netherlands | retrospective cohort | 2201 | 371 | 1830 | NA | NA | NA | NA | 1.16 (1.06,1.26) | Available | meta-analysis |

**Table S2-3 *ZNF512B* rs2275294**

| ***ZNF512B* rs2275294 CC+CT vs TT** | | | | | | | | | | | | | |
| --- | --- | --- | --- | --- | --- | --- | --- | --- | --- | --- | --- | --- | --- |
| First author | Year | Country/region | Type of study | Total ALS patients | Number of CC+CT carriers | Number of TT  carriers | Median or mean survival from onset | | Diagnosis | | HR (95%CI) | HR source | systematic review or pairwise meta-analysis |
|  |  |  |  |  |  |  | CC+CT carriers | TT carriers | criteria | level |  |  |  |
| S. Tetsuka | 2012 | Japan | retrospective case-control | 176 | 128 | 48 | Mean ±SD:2.03±1.15 | Mean ±SD: 3.05±1.64 | revised El Escorial criteria | definite ALS | 1.807(1.018,3.209) | Available | meta-analysis |
| C. J. Yu | 2018 | China | prospective cohort | 59 | 39 | 20 | NA | NA | both clinical symptoms, signs and electromyography (EMG) positive manifestation | NA | 2.587(1.161,5.765) | Available | meta-analysis |
| H. Jiang | 2021 | China | retrospective case-control | 212 | 166 | 46 | NA | NA | revised El Escorial criteria | NA | 1.98(1.21,3.24) | Available | meta-analysis |

**Table S2-4 *KIFAP3* rs1541160**

| ***KIFAP3* rs1541160 CC vs CT vs TT** | | | | | | | | | | | | | | | |
| --- | --- | --- | --- | --- | --- | --- | --- | --- | --- | --- | --- | --- | --- | --- | --- |
| First author | Year | Country/region | Type of study | Total ALS patients | Number of CC carriers | Number of CT carriers | Number of TT  carriers | Median or mean survival from onset | | | Diagnosis | | HR (95%CI) | HR source | systematic review or pairwise meta-analysis |
|  |  |  |  |  |  |  |  | CC carriers | CT carriers | TT carriers(ref) | criteria | level |  |  |  |
| J. E. Landers | 2009 | England,France, Netherlands, USA | retrospective case-control | 1014 | 100 | 403 | 511 | median3.96y | median 2.84y | median 2.67y | revised El Escorial criteria | probable or definite ALS | CC: 0.78(0.62,0.99)  CT: 0.91(0.8,1.04) | Estimated | meta-analysis |
| B. J. Traynor | 2010 | northwestern Italy | retrospective case-control | 504 | 39 | 233 | 232 | median 4.09y, 95%CI (2.69，NA) | median 3.48y, 95%CI (3.15,3.94) | median 3.58y, 95%CI (3.12,4.33) | revised El Escorial criteria | probable or definite ALS | CC: 1.12(0.79,1.59)  CT: 1.15(0.95,1.38)  CC+CT: 1.17(0.98,1.39) | Estimated | meta-analysis for CC, CT. systematic review for CC+CT. |
| V. Orsetti | 2011 | Padua, Italy | retrospective case-control | 109 | 15 | 83 | 111 | median 4.0y, 95% CI (1.78,6.21) | median 4.92y ,95% CI (3.27,5.80) | median 4.0y, 95%CI (2.45,5.54) | revised El Escorial criteria | NA | CC: 1.62(0.95,2.78)  CT:0.85(0.61,1.19) | Estimated | meta-analysis |
| P. T. van Doormaal | 2014 | Belgium, Germany, Ireland, Italy, Netherlands, and Sweden | retrospective case-control | 2261 | 192 | 949 | 1120 | median2.6y; mean3.14y | median2.39y; mean3.01y | median 2.4y; mean 3.13y | revised El Escorial criteria | possible ALS or higher | CC: 0.87(0.72,1.04)  CT: 1.1(0.92,1.12)  **TT+CT vs CC (**ref): 0.86(0.72,1.03) | Available | meta-analysis for CC, CT.  systematic review for TT+CT. |
| D. Czell | 2017 | Switzerland and Sweden | retrospective case-control | 71 | 6 | 31 | 34 | median 2.43y | median 3.82y | median 3.27y | revised El Escorial criteria | probable or definite ALS | CC: 1.38(0.61,3.15)  CT:0.89(0.55,1.44) | Estimated | meta-analysis |
| I. Fogh | 2016 | Europe and USA | retrospective case-control | 4256 | NA | NA | NA | NA | NA | NA | El Escorial revised criteria | NA | **CC+CT vs TT** (ref): 1.04(0.98,1.1) | Available | systematic review |

**Table S3 Characteristics of the articles included in systematic review for other modification loci.**

| ***SMN2* with deletion vs without deletion** | | | | | | | | | | | | | | | | | |
| --- | --- | --- | --- | --- | --- | --- | --- | --- | --- | --- | --- | --- | --- | --- | --- | --- | --- |
| First author | Year | Country/region | Type of study | Total ALS patients | patients with deletion | | patients without deletion | | Median or mean survival from onset | | | | Diagnosis | | HR (95%CI) | HR source | systematic review or pairwise meta-analysis |
|  |  |  |  |  |  |  |  |  | with deletion | | | without deletion  (ref) | criteria | level |  |  |  |
| J. Gamez | 2002 | Spain | retrospective case-control | 124 | 11 | | 113 | | NA | | | NA | El Escorial criteria | NA | 1.46 (0.8,2.66) | Estimated | systematic review |
| ***CX3CR1* V249I VI+II vs VV** | | | | | | | | | | | | | | | | | |
| First author | Year | Country/region | Type of study | Total ALS patients | patients with V249I VI+II | | patients with V249I II | | Median or mean survival from onset | | | | Diagnosis | | HR (95%CI) | HR source | systematic review or pairwise meta-analysis |
|  |  |  |  |  |  |  |  |  | V249I VI+II | | | V249I VV (ref) | criteria | level |  |  |  |
| A. Lopez-Lopez (sALS cohort) | 2014 | Spain | retrospective case-control | 107 | 51 | | 56 | | median ± SEM 5.63y±0.62 | | | median ± SEM 3.52y±0.41 | revised El Escorial criteria | Laboratory supported or definite ALS | 1.72 (1.15,2.67) | Available | systematic review |
| A. Calvo | 2018 | Italy | retrospective case-control | 755 | 387 | | 368 | | median 3.1y, IQR (1.7-5.3) | | | median 2.7y, IQR (1.7-4.4) | revised El Escorial criteria | definite, probable, and probable laboratory-supported ALS | 1.24 (1.07,1.44) | Estimated | systematic review |
| ***CX3CR1* T280M MM vs MT vs TT** | | | | | | | | | | | | | | | | | |
| First author | Year | Country/region | Type of study | Total ALS patients | patients with T280M MM | patients with T280M MT | patients with T280M TT | | Median or mean survival from onset | | | | Diagnosis | | HR (95%CI) | HR source | systematic review or pairwise meta-analysis |
|  |  |  |  |  |  |  |  |  | MM | | MT | TT (ref) | criteria | level |  |  |  |
| A. Calvo | 2018 | Italy | retrospective case-control | 755 | 22 | 175 | 558 | | median 3.7y, IQR (1.9-2.8) | | median 2.8y, IQR (1.8-5.6) | median 2.7y, IQR (1.8-4.6) | revised El Escorial criteria | definite, probable, and probable laboratory-supported ALS | MM: 1.24 (0.78,1.97)  MT: 1.1 (0.92,1.31) | Estimated | systematic review |
| ***CX3CR1* 249V/V + 280T/T or 249V/I and 280T/M or 249V/V + 280M/M or 249I/I + 280T/T vs V249I II & T280M MM + V249 II & T280 TM + V249I VI and T280M MM (ref)** | | | | | | | | | | | | | | | | | |
| First author | Year | Country/region | Type of study | Total ALS patients | patients with the former haplotype | | patients with the later haplotype | | Median or mean survival from onset | | | | Diagnosis | | HR (95%CI) | HR source | systematic review or pairwise meta-analysis |
|  |  |  |  |  |  |  |  |  | the former haplotype | | | the later haplotype | criteria | level |  |  |  |
| A. Calvo | 2018 | Italy | retrospective case-control | 755 | 42 | | 713 | | NA | | | NA | revised El Escorial criteria | definite, probable, and probable laboratory-supported ALS | 0.69 (0.46,0.93) | Available | systematic review |
| ***ABCC8* rs4148646 GG vs GC+CC** | | | | | | | | | | | | | | | | | |
| First author | Year | Country/region | Type of study | Total ALS patients | patients with GG carriers | | patients with GG+GC carriers | | Median or mean survival from onset | | | | Diagnosis | | HR (95%CI) | HR source | systematic review or pairwise meta-analysis |
|  |  |  |  |  |  |  |  |  | GG carriers | | | GC+CC carriers(ref) | criteria | level |  |  |  |
| J. M. Vidal-Taboada(bublar onset cohort) | 2018 | Spain | retrospective case-control | NA | NA | | NA | | median 12.8y, 95% CI 0, 33.4) | | | median 2.0y, 95% CI (1.21, 2.94) | revised El Escorial criteria | NA | 0.25 (0.07,0.86) | Available | systematic review |
| ***KCNJ11* rs5219 TT vs CT+CC** | | | | | | | | | | | | | | | | | |
| First author | Year | Country/region | Type of study | Total ALS patients | patients with TT carriers | | patients with CT+CC carriers | | Median or mean survival from onset | | | | Diagnosis | | HR (95%CI) | HR source | systematic review or pairwise meta-analysis |
|  |  |  |  |  |  |  |  |  | TT carriers | | | CT+CC carriers | criteria | level |  |  |  |
| J. M. Vidal-Taboada (bublar onset cohort) | 2018 | Spain | retrospective case-control | NA | NA | | NA | | median 12.8y, 95% CI (0, 33.2) | | | median 2.0y, 95% CI (0.83, 3.17) | revised El Escorial criteria | NA | 0.31 (0.1,0.91) | Available | systematic review |
| ***LXRs rs2279238* TT vs CT vs CC** | | | | | | | | | | | | | | | | | |
| First author | Year | Country/region | Type of study | Total ALS patients | patients with TT carriers | patients with CT carriers | patients with CC carriers | | Median or mean survival from onset | | | | Diagnosis | | HR (95%CI) | HR source | systematic review or pairwise meta-analysis |
|  |  |  |  |  |  |  |  |  | TT carriers | | CT carriers | CC carriers(ref) | criteria | level |  |  |  |
| K. Mouzat | 2017 | France | retrospective case-control | 438 | 11 | 104 | 323 | | NA | | NA | NA | revised El Escorial criteria | definite or probable ALS | TT: 1.64 (0.89,3.05)  CT: 1.27 (0.99,1.56) | Estimated | systematic review |
| ***LXRs* rs7120118 CC vs CT vs TT** | | | | | | | | | | | | | | | | | |
| First author | Year | Country/region | Type of study | Total ALS patients | patients with CC carriers | patients with CT carriers | patients with TT carriers | | Median or mean survival from onset | | | | Diagnosis | | HR (95%CI) | HR source | systematic review or pairwise meta-analysis |
|  |  |  |  |  |  |  |  |  | CC  carriers | | CT carriers | TT carriers(ref) | criteria | level |  |  |  |
| K. Mouzat | 2017 | France | retrospective case-control | 438 | 15 | 182 | 241 | | NA | | NA | NA | revised El Escorial criteria | definite or probable ALS | CC: 1.66 (0.97,2.84)  CT: 0.97 (0.81,1.18) | Estimated | systematic review |
| ***LXRs* rs35463555 AA vs AG vs GG** | | | | | | | | | | | | | | | | | |
| First author | Year | Country/region | Type of study | Total ALS patients | patients with AA carriers | patients with AG carriers | patients with GG carriers | | Median or mean survival from onset | | | | Diagnosis | | HR (95%CI) | HR source | systematic review or pairwise meta-analysis |
|  |  |  |  |  |  |  |  |  | AA  carriers | | AG carriers | GG carriers(ref) | criteria | level |  |  |  |
| K. Mouzat | 2017 | France | retrospective case-control | 438 | 50 | 181 | 207 | | NA | | NA | NA | revised El Escorial criteria | definite or probable ALS | AA: 0.88 (0.64,1.2)  AG: 1.05 (0.86,1.28) | Estimated | systematic review |
| ***LXRs* rs2695121 TT vs CT vs CC** | | | | | | | | | | | | | | | | | |
| First author | Year | Country/region | Type of study | Total ALS patients | patients with TT carriers | patients with CT  carriers | patients with CC carriers | | Median or mean survival from onset | | | | Diagnosis | | HR (95%CI) | HR source | systematic review or pairwise meta-analysis |
|  |  |  |  |  |  |  |  |  | TT  carriers | | CT  carriers | CC carriers(ref) | criteria | level |  |  |  |
| K. Mouzat | 2017 | France | retrospective case-control | 438 | 72 | 224 | 142 | | median 2.47y, IQR(1.65,4.03) | | median 3.12y, IQR (2,5.5) | median 2.9y, IQR (1.71–5.82) | revised El Escorial criteria | definite or probable ALS | TT: 1.46 (1.24,1.99)  CT: 0.98 (0.78,1.24)  TT+CT:1.47(1.12,1.93) | Available | systematic review |
| ***PRGN* IVS2+21G>A (****rs9897526 GG vs GA+AA)** | | | | | | | | | | | | | | | | | |
| First author | Year | Country/region | Type of study | Total ALS patients | patients with GG carriers | patients with GA+AA carriers | | | Median or mean survival from onset | | | | Diagnosis | | HR (95%CI) | HR source | systematic review or pairwise meta-analysis |
|  |  |  |  |  |  |  |  |  | GG carriers | | | GA+AA carriers(ref) | criteria | level |  |  |  |
| K. Sleegers(Belgium cohort) | 2008 | Belgium | retrospective case-control | 230 | NA | NA | | | NA | | | NA | revised El Escorial criteria | definite, probable, or laboratory supported probable ALS | 1.7 (1.1,2.64) | Available | systematic review |
| ***PRGN***  **IVS3-47-46insGTCA and IVS4+24G>A (** **rs34424835 and rs850713)** | | | | | | | | | | | | | | | | | |
| First author | Year | Country/region | Type of study | Total ALS patients | patients with rare allele | patients without rare allele | | | Median or mean survival from onset | | | | Diagnosis | | HR (95%CI) | HR source | systematic review or pairwise meta-analysis |
|  |  |  |  |  |  |  |  |  | with rare allele | | | without rare allele(ref) | criteria | level |  |  |  |
| K. Sleegers(Belgium cohort) | 2008 | Belgium | retrospective case-control | 230 | NA | NA | | | NA | | | NA | revised El Escorial criteria | definite, probable, or laboratory supported probable ALS | 1.82 (0.84,3.95) | Available | systematic review |
| ***PRGN* IVS3-47-46insGTCA (rs34424835)** | | | | | | | | | | | | | | | | | |
| First author | Year | Country/region | Type of study | Total ALS patients | patients with rare allele | patients without rare allele | | | Median or mean survival from onset | | | | Diagnosis | | HR (95%CI) | HR source | systematic review or pairwise meta-analysis |
|  |  |  |  |  |  |  |  |  | with rare allele | | | without rare allele(ref) | criteria | level |  |  |  |
| K. Sleegers(Dutch cohort) | 2008 | Netherlands | retrospective case-control | 308 | NA | NA | | | NA | | | NA | revised El Escorial criteria | NA | 2.29 (1.15,4.55) | Available | systematic review |
| ***HTR2B* rs10199752 AA vs AC+CC** | | | | | | | | | | | | | | | | | |
| First author | Year | Country/region | Type of study | Total ALS patients | patients with AA | patients with AC+CC | | | Median or mean survival from onset | | | | Diagnosis | | HR (95%CI) | HR source | systematic review or pairwise meta-analysis |
|  |  |  |  |  |  |  |  |  | AA carriers | | | AC+CC carriers(ref) | criteria | level |  |  |  |
| H. El Oussini | 2016 | Netherlands | retrospective case-control | 1677 | 759 | 918 | | | NA | | | NA | revised El Escorial criteria | probable or definite ALS | 0.88 (0.79,0.98) | Available | systematic review |
| ***STMN2* CA repeat long/long 24 CA vs other** | | | | | | | | | | | | | | | | | |
| First author | Year | Country/region | Type of study | Total ALS patients | patients with long/long 24 CA | patients with other genotypes | | | Median or mean survival from onset | | | | Diagnosis | | HR (95%CI) | HR source | systematic review or pairwise meta-analysis |
|  |  |  |  |  |  |  |  |  | long/long 24 CA | | | other genotypes (ref) | criteria | level |  |  |  |
| F. Theunissen  (USA cohort) | 2021 | USA | retrospective case-control | 143 | 37 | 106 | | | NA | | | NA | revised El Escorial criteria | NA | 1.21 (0.82,1.78) | Available | systematic review |
| F. Theunissen  (Australian cohort) | 2021 | Australian | prospective cohort | 67 | 41 | 26 | | | NA | | | NA | revised El Escorial criteria | NA | 1.79 (0.62,5.2) | Available | systematic review |
| ***BDNF*** ***C270T CT* vs. *CC*** | | | | | | | | | | | | | | | | | |
| First author | Year | Country/region | Type of study | Total ALS patients | patients with CT | patients with CC | | | Median or mean survival from onset | | | | Diagnosis | | HR (95%CI) | HR source | systematic review or pairwise meta-analysis |
|  |  |  |  |  |  |  |  |  | CT carriers | | | CC carriers(ref) | criteria | level |  |  |  |
| Lianping Xu | 2017 | China | retrospective cohort | 499 | 53 | 446 | | | NA | | | NA | El Escorial criteria | definite or probable ALS | 1.28 (0.84,1.96) | Estimated | systematic review |
| ***C7* gene rs3792646 AA vs AC** | | | | | | | | | | | | | | | | | |
| First author | Year | Country/region | Type of study | Total ALS patients | patients with AA | patients with AC + CC | | | Median or mean survival from onset | | | | Diagnosis | | HR (95%CI) | HR source | systematic review or pairwise meta-analysis |
|  |  |  |  |  |  |  |  |  | AA carriers | | | AC carriers(ref) | criteria | level |  |  |  |
| Ji He | 2020 | China | prospective cohort | 262 | 22 | 240 | | | mean ± SD 2.56y ± 0.25 | | | mean ± SD 3.40y ± 0.13 | revised El Escorial criteria | possible, probable, or definite ALS | 0.427 (0.238,0.768) | Available | systematic review |
| ***PON1* L55M (rs854560) MM vs LM vs LL** | | | | | | | | | | | | | | | | | |
| First author | Year | Country/region | Type of study | Total ALS patients | patients with MM | patients with LM | | patients with LL | Median or mean survival from onset | | | | Diagnosis | | HR (95%CI) | HR source | systematic review or pairwise meta-analysis |
|  |  |  |  |  |  |  |  |  | MM carriers | LM carriers | | LL carriers(ref) | criteria | level |  |  |  |
| F. P. Diekstra  (Rural cohort) | 2009 | England | retrospective case-control | 49 | 12 | 19 | | 18 | NA | NA | | NA | NA | NA | MM: 2.83 (1.6,1.67)  LM: 1.93 (0.85,4.35) | Estimated | systematic review |
| F. P. Diekstra  (Urban cohort) | 2009 | England | retrospective case-control | 49 | 0 | 24 | | 25 | NA | NA | | NA | NA | NA | LM: 0.75 (0.41,1.37) | Estimated | systematic review |
| ***PON1* rs662 GG+GA vs AA** | | | | | | | | | | | | | | | | | |
| First author | Year | Country/region | Type of study | Total ALS patients | patients with GG+GA | patients with AA | | | Median or mean survival from onset | | | | Diagnosis | | HR (95%CI) | HR source | systematic review or pairwise meta-analysis |
|  |  |  |  |  |  |  |  |  | GG+GA carriers | | | AA carriers(ref) | criteria | level |  |  |  |
| F. Verde | 2019 | Italy | prospective cohort | 409 | NA | NA | | | Mean 3.71y | | | Mean 4.21y | revised El Escorial criteria | NA | 1.38(1.07,1.77) | Available | systematic review |
| ***NIPA1* polyalanine repeat expansions long vs short vs** **normal** | | | | | | | | | | | | | | | | | |
| First author | Year | Country/region | Type of study | Total ALS patients | patients with long expansions | patients with short expansions | patients with normal expansions | | Median or mean survival from onset | | | | Diagnosis | | HR (95%CI) | HR source | systematic review or pairwise meta-analysis |
|  |  |  |  |  |  |  |  |  | long expansions | | short expansions | normal expansions(ref) | criteria | level |  |  |  |
| H. M. Blauw  (the Netherlands cohort) | 2012 | Netherlands | retrospective case-control | 924 | 59 | 10 | 855 | | median 2.83y, SD1.78 | | median 4.39y, SD 1.08 | median 2.78y, SD 3.34 | the 1994 El Escorial criteria | probable or definite  ALS | Long: 1.73 (1.3,2.32)  Short: 0.67 (0.32,1.41) | Available | systematic review |
| H. M. Blauw  (Belgium cohort) | 2012 | Belgium | retrospective case-control | 362 | 16 | 1 | 345 | | median 2.0y, SD NA | | median 2.5y, SD2.37 | median 2.98y, SD1.50 | the 1994 El Escorial criteria | probable or definite  ALS | Long:1.16 (0.66,2.06)  Short:2.75 (0.38,19.77) | Available | systematic review |
| ***SLC11A2* rs407135 C carriers vs non-C carriers** | | | | | | | | | | | | | | | | | |
| First author | Year | Country/region | Type of study | Total ALS patients | patients with C allele | patients without C allele | | | Median or mean survival from onset | | | | Diagnosis | | HR (95%CI) | HR source | systematic review or pairwise meta-analysis |
|  |  |  |  |  |  |  |  |  | C carriers | | | non-C carriers | criteria | level |  |  |  |
| H. Blasco | 2011 | France | prospective case-control | 197 | 113 | 84 | | | median 4.15y, range (0.23,29.8) | | | median 5.61y, range (0.57-26.67) | Airlie House | NA | 1.5 (1.1,2.1) | Available | systematic review |
| ***CAMTA1 s2412208 GG+GT vs TT*** | | | | | | | | | | | | | | | | | |
| First author | Year | Country/region | Type of study | Total ALS patients | patients with GG + GT | patients with TT | | | Median or mean survival from onset | | | | Diagnosis | | HR (95%CI) | HR source | systematic review or pairwise meta-analysis |
|  |  |  |  |  |  |  |  |  | GG + GT carriers | | | TT carriers | criteria | level |  |  |  |
| I. Fogh | 2016 | Europe and USA | retrospective case-control | 4256 | NA | NA | | | median 3.05y, 95%CI (2.93,3.17) | | | median 3.4y, 95%CI (2.56,3.54) | revised El Escorial criteria | NA | 1.18 (1.09,1.26) | Available | systematic review |
| ***GSTP1* rs1695 GG vs GA vs AA** | | | | | | | | | | | | | | | | | |
| First author | Year | Country/region | Type of study | Total ALS patients | patients with GG | patients with GA | patients with AA | | Median or mean survival from onset | | | | Diagnosis | | HR (95%CI) | HR source | systematic review or pairwise meta-analysis |
|  |  |  |  |  |  |  |  |  | GG carriers | | GA carriers | AA carriers(ref) | criteria | level |  |  |  |
| J. B. S. Barros | 2021 | Brazil | retrospective case-control | 101 | 6 | 42 | 53 | | NA | | NA | NA | the laboratory and imaging guidelines | regardless of the stage of the disease | GG: 0.52 (0.16,1.62)  GA: 0.66 (0.42,1.05) | Estimated | systematic review |
| ***CNTF* MM vs NM vs NN (The wild-type allele is coded as N, the null mutant as M)** | | | | | | | | | | | | | | | | | |
| First author | Year | Country/region | Type of study | Total ALS patients | patients with MM | patients with NM | patients with NN | | Median or mean survival from onset | | | | Diagnosis | | HR (95%CI) | HR source | systematic review or pairwise meta-analysis |
|  |  |  |  |  |  |  |  |  | MM carriers | | NM carriers | NN carriers(ref) | criteria | level |  |  |  |
| A. Al-Chalabi | 2003 | USA, UK, Scandinavia | retrospective case-control | 231 | 5 | 53 | 5 | | median 2.58y | | median 3.42y | median 3.0y | NA | NA | MN: 1.13 (0.82,1.57)  NN: 2.33 (0.75,7.28) | Estimated | systematic review |
| ***CNTF* splice site mutation AA vs GA vs GG** | | | | | | | | | | | | | | | | | |
| First author | Year | Country/region | Type of study | Total ALS patients | patients with AA | patients with GA | patients with GG | | Median or mean survival from onset | | | | Diagnosis | | HR (95%CI) | HR source | systematic review or pairwise meta-analysis |
|  |  |  |  |  |  |  |  |  | AA carriers | | GA carriers | GG carriers(ref) | criteria | level |  |  |  |
| P. W. Van Vught | 2007 | Netherlands | retrospective case-control | NA | NA | NA | NA | | NA | | NA | NA | revised El Escorial criteria | Probable and definite ALS | AA: 1.14 (0.8,1.64)  GA: 0.74 (0.32,1.72) | Available | systematic review |
| ***HLA-DRA or HLA-DRB5* rs9268856 AA vs AC + CC** | | | | | | | | | | | | | | | | | |
| First author | Year | Country/region | Type of study | Total ALS patients | patients with AA | patients with AC+CC | | | Median or mean survival from onset | | | | Diagnosis | | HR (95%CI) | HR source | systematic review or pairwise meta-analysis |
|  |  |  |  |  |  |  |  |  | AA carriers | | | AC+CC carriers(ref) | criteria | level |  |  |  |
| X. Yang | 2017 | China | retrospective cohort | 400 | 33 | 367 | | | mean 3.08y ± 1.66 | | | mean 2.07y ± 1.35 | revised El Escorial criteria | NA | 2.431 (1.55,43.83) | Available | systematic review |
| **rs4623951** **CC vs AC vs AA** | | | | | | | | | | | | | | | | | |
| First author | Year | Country/region | Type of study | Total ALS patients | patients with AA | patients with AC | patients with CC | | Median or mean survival from onset | | | | Diagnosis | | HR (95%CI) | HR source | systematic review or pairwise meta-analysis |
|  |  |  |  |  |  |  |  |  | AA carriers | | AC carriers | CC carriers | criteria | level |  |  |  |
| I. Fogh | 2016 | Europe, USA | retrospective case-control | 4256 | NA | NA | NA | | NA | | NA | NA | revised El Escorial criteria | NA | additive genetic model: 1.07 (1.03,1.13) | Available | systematic review |
| ***EPHA4* rs6436254 AA vs GA vs GG** | | | | | | | | | | | | | | | | | |
| First author | Year | Country/region | Type of study | Total ALS patients | patients with AA | patients with GA | patients with GG | | Median or mean survival from onset | | | | Diagnosis | | HR (95%CI) | HR source | systematic review or pairwise meta-analysis |
|  |  |  |  |  |  |  |  |  | AA carriers | | AC carriers | CC carriers(ref) | criteria | level |  |  |  |
| I. Fogh | 2016 | Europe, USA | retrospective case-control | 4256 | NA | NA | NA | | NA | | NA | NA | revised El Escorial criteria | NA | additive genetic model: 1.07 (1.02,1.26) | Available | systematic review |
| ***IDE* rs139550538 AA/AT/TT** | | | | | | | | | | | | | | | | | |
| First author | Year | Country/region | Type of study | Total ALS patients | patients with AA | patients with AT | patients with TT | | Median or mean survival from onset | | | | Diagnosis | | HR (95%CI) | HR source | systematic review or pairwise meta-analysis |
|  |  |  |  |  |  |  |  |  | AA carriers | | AT  carriers | TT carriers(ref) | criteria | level |  |  |  |
| I. Fogh | 2016 | Europe, USA | retrospective case-control | 4256 | 2 | 224 | 4030 | | 19 | | 31 | 39 | revised El Escorial criteria | NA | additive genetic model: 1.61 (1.38,1.89)  AA+AT:1.52(1.31,1.77) | Available | systematic review |
| ***SMN1* 4 vs 3 vs 2 vs 1 copy numbers**   \| First author \| Year \| Country/region \| Type of study \| Total ALS patients \| Number of 4 copies carriers \| Number of 3 copies carriers \| Number of 2 copies carriers \| Number of 1 copy carriers \| Median or mean survival from onset \| \| \| \| Diagnosis \| \| HR (95%CI) \| HR source \| systematic review or pairwise meta-analysis \| \| --- \| --- \| --- \| --- \| --- \| --- \| --- \| --- \| --- \| --- \| --- \| --- \| --- \| --- \| --- \| --- \| --- \| --- \| \| 4 copies \| 3 copies \| 2 copies \| 1 copy  (ref) \| criteria \| level \| \| M. Moisse \| 2021 \| Europe (Project MinE) \| retrospective case-control \| 5872 \| 20 \| 364 \| 5731 \| 117 \| NA \| NA \| NA \| NA \| revised El Escorial criteria \| NA \| 4 copies: 1.53(0.93,2.51)  3 copies: 0.93(0.74,1.16)  2 copies: 0.91(0.75,1.1) \| Estimated \| systematic review; \| | | | | | | | | | | | | | | | | | |
| ***SMN2* 4 vs 3 vs 2 vs 1 vs 0 copy numbers**   \| First author \| Year \| Country/region \| Type of study \| Total ALS patients \| Number of 3/4 copies carriers \| Number of 2 copies carriers \| Number of 1 copy carriers \| Number of 0 copy carriers \| Median or mean survival from onset \| \| \| \| \| Diagnosis \| \| HR (95%CI) \| HR source \| systematic review or pairwise meta-analysis \| \| --- \| --- \| --- \| --- \| --- \| --- \| --- \| --- \| --- \| --- \| --- \| --- \| --- \| --- \| --- \| --- \| --- \| --- \| --- \| \| 4 copies \| 3 copies \| 2 copies \| 1 copy \| 0 copy(ref) \| criteria \| level \| \| M. Moisse \| 2021 \| Europe (Project MinE) \| retrospective case-control \| 5872 \| 178 \| 2855 \| 2363 \| 476 \| NA \| NA \| NA \| NA \| NA \| revised El Escorial criteria \| NA \| 3/4 copies: 1.06(0.86,1.28)  2 copies: 1.06(0.96,1.17)  1 copy: 1.08(0.98,1.2) \| Estimated \| systematic review \| | | | | | | | | | | | | | | | | | |

‘Estimated’: HR was evaluated from Kaplan-Meier curve or other curves. ‘Available’: HR was reported in the article. ALS: amyotrophic lateral sclerosis; HR: hazard ratio; CI: confidence interval; SD: standard deviation; NA: not available.

**Table** **S4 The quality of articles included in network meta-analysis and pairwise meta-analysis by NOS.**

| **Network meta-analysis (n=35)** | | | | |
| --- | --- | --- | --- | --- |
| **Study ID** | **Selection (4’)** | **Comparability (2’)** | **Exposure/outcome measure (3’)** | **NOS score** |
| G. Borghero (2015) | ☆☆☆ | ☆ | ☆☆ | 6 |
| A. Chiò (2015) | ☆☆☆ | ☆ | ☆☆☆ | 7 |
| S. Byrne (2012) | ☆☆ | ☆ | ☆☆ | 5 |
| A. Ratti (2012) | ☆☆☆ | ☆ | ☆☆ | 6 |
| M. Sabatelli (2012) | ☆☆☆ | ☆ | ☆☆☆ | 7 |
| W. van Rheenen(2012) | ☆☆☆☆ | ☆ | ☆☆☆ | 8 |
| S. Millecamps (2012) | ☆☆☆ | ☆ | ☆☆☆ | 7 |
| S. Debray (2013) | ☆☆☆☆ | ☆☆ | ☆☆☆ | 9 |
| A. García-Redondo (2012) | ☆☆☆ | ☆ | ☆☆☆ | 7 |
| D. J. Irwin (2013) | ☆☆ | ☆ | ☆☆☆ | 6 |
| K. Van Laere (2014) | ☆☆ | ☆☆ | ☆☆ | 6 |
| A. Calvo (2016) | ☆☆☆ | ☆ | ☆☆☆ | 7 |
| R. Govaarts (2016) | ☆☆☆ | ☆ | ☆☆☆ | 7 |
| M. E. Umoh (2016) | ☆☆☆ | ☆☆ | ☆☆☆ | 8 |
| T. F. Gendron (2017) | ☆☆ | ☆ | ☆☆☆ | 6 |
| W. Reniers (2017) | ☆☆ | ☆ | ☆☆ | 5 |
| G. Miltenberger-Miltenyi (2019) | ☆☆ | ☆ | ☆☆ | 5 |
| A. J. Cammack (2019) | ☆☆☆☆ | ☆☆ | ☆☆☆ | 9 |
| J. Rooney (2019) | ☆☆ | ☆ | ☆☆☆ | 6 |
| F. Trojsi (2019) | ☆☆☆ | ☆☆ | ☆☆☆ | 7 |
| M. Benatar (2020) | ☆☆ | ☆ | ☆☆ | 5 |
| M. De Schaepdryver (2020) | ☆☆ | ☆ | ☆☆ | 5 |
| D. Brand (2021) | ☆☆ | ☆ | ☆☆☆ | 6 |
| U. Kläppe (2021) | ☆☆☆ | ☆ | ☆☆☆ | 7 |
| F. Puentes (2021) | ☆☆ | ☆ | ☆ | 4 |
| M. E. Cudkowicz (1997) | ☆☆ | ☆ | ☆ | 4 |
| A. Hübers (2015) | ☆☆☆ | ☆ | ☆☆☆ | 7 |
| P. Corcia (2012) | ☆☆☆ | ☆ | ☆☆☆ | 7 |
| W. Liu (2021) | ☆☆☆ | ☆ | ☆☆☆ | 7 |
| S. Lattante (2012) | ☆☆☆☆ | ☆ | ☆☆☆ | 8 |
| G. Borghero (2014) | ☆☆ | ☆ | ☆☆☆ | 6 |
| S. Millecamps (2010) | ☆☆☆ | ☆ | ☆☆☆ | 7 |
| A. Chiò (2012) | ☆☆☆ | ☆☆ | ☆☆☆ | 8 |
| E. P. McCann (2017) | ☆☆ | ☆ | ☆☆ | 5 |
| Y. P. Chen (2021) | ☆☆☆ | ☆ | ☆☆☆ | 7 |
| **Pairwise meta-analysis(n=20)** | | | | |
| **Study ID** | **Selection (4’)** | **Comparability (2’)** | **Exposure/outcome measure (3’)** | **NOS score** |
| A. Al-Chalabi (1996) | ☆☆ | ☆ | ☆☆ | 5 |
| V. E. Drory (2001) | ☆☆☆ | ☆ | ☆☆ | 6 |
| H. Zetterberg (2008) | ☆☆☆ | ☆ | ☆☆☆ | 7 |
| K. Steenland (2010) | ☆☆ | ☆ | ☆ | 4 |
| F. P. Diekstra (2012) | ☆☆ | ☆ | ☆☆ | 5 |
| A. Chiò (2013) | ☆☆☆ | ☆ | ☆☆☆ | 7 |
| J. M. Vidal-Taboada (2015) | ☆☆☆ | ☆ | ☆☆☆ | 7 |
| I. Fogh (2016) | ☆☆ | ☆ | ☆☆ | 5 |
| H. H. G. Tan (2020) | ☆☆☆ | ☆ | ☆☆☆ | 7 |
| S. Tetsuka (2012) | ☆☆☆ | ☆ | ☆☆ | 6 |
| C. J. Yu (2018) | ☆☆ | ☆ | ☆☆ | 5 |
| H. Jiang (2021) | ☆☆☆ | ☆ | ☆☆ | 6 |
| J. E. Landers (2009) | ☆☆☆ | ☆ | ☆☆☆ | 7 |
| B. J. Traynor (2010) | ☆☆☆ | ☆☆ | ☆☆☆ | 8 |
| V. Orsetti (2011) | ☆☆☆ | ☆ | ☆☆☆ | 7 |
| P. T. van Doormaal (2014) | ☆☆☆ | ☆ | ☆☆☆ | 7 |
| D. Czell (2017) | ☆☆☆ | ☆ | ☆☆☆ | 7 |

NOS：Newcastle-Ottawa Scale

**Table S5** **The feathers for different variants in *SOD1*, *FUS*, *TARDBP*.**

| Gene | Variants | Position(hg19) | HGVS | Effect | Survival |
| --- | --- | --- | --- | --- | --- |
| *SOD1* | A4V | Chr21:33032096 | NM_000454.4:  c.14C>T | missense + splice_region | Mean disease duration of SOD1^A4V^ ALS (year) ± SD:1.4±0.7, significantly decreased compared with SOD1^non-A4V^ (6.6±7.0). |
|  | H46R | Chr21:33036170 | NM_000454.4:  c.140A>G | missense | Mean disease duration of SOD1^H46R^ ALS (year) ± SD:12±7.6. |
|  | D90A | Chr21:33036155 | NM_000454.4:  c.272A>C | missense | Mean disease duration of SOD1^G41A^ ALS (year) ± SD: 9.7±5.4. |
|  | I113T | Chr21:33039672 | NM_000454.4:  c.341T>C | missense | Mean disease duration of SOD1^I113T^ ALS (year) ± SD: 5.3±4.8. |
| *FUS* | P525L | Chr16:31202752 | NM_004960.3:  c.1574C>T | missense | Median OTSE (year) (95%CI): 1.08 (0.83–1.67) |
|  | R521 mutations | NA | NA | missense | Median OTSE (year) (95%CI): 2.58 (2.08–3.33) |
|  | frameshift/truncation | NA | NA | frameshift/ nonsense | Median OTSE (year) (95%CI): 1.58 (1.04–2.17) |
| *TARDBP* | G348C | Chr1: 11082508 | NM_007375.3:  c.1042G>T | missense | Median (year), (range) 6.78 (3.0–13) |
|  | A382T | Chr1: 11082610 | NM_007375.3:c.1144G>A | missense | Median (year), (range) 4.84 (1.42–15) |
|  | M337V | Chr1: 11082475 | NM_007375.3:  c.1009A>G | missense | Median (year), (range) 8.75 (1.0–17) |

HGVS：Human Genome Variation Society; SD：Standard Deviation; NA: Not Applicable. OTSE：survival time until severe event; CI：Confidence interval;

**Figure S1 forest plot for inconsistency test in network meta-analysis.**

**
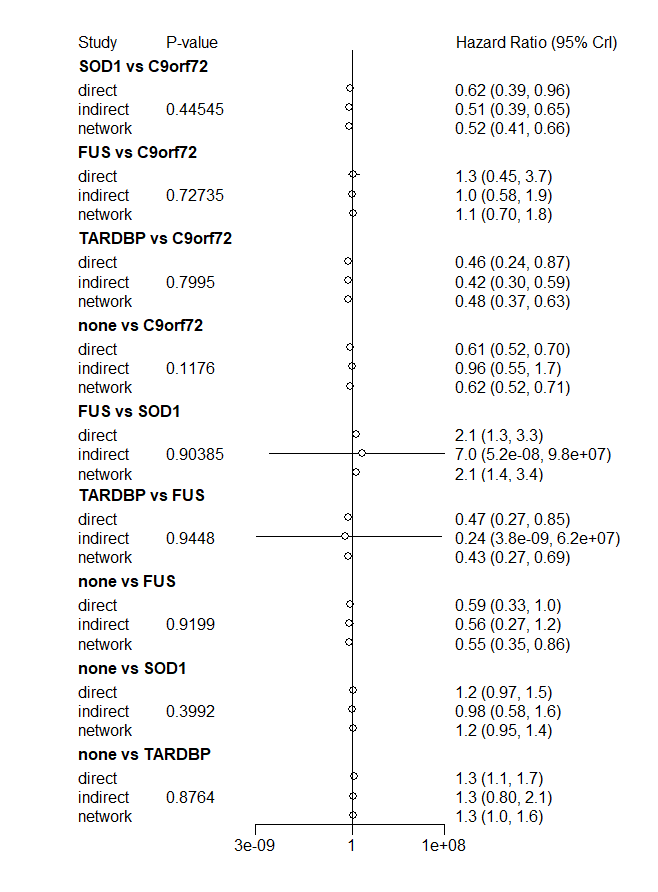
**

**Figure S2 forest plot for heterogeneity test for none vs *ATXN2*, *CCNF* vs *C9orf72*, *FUS* vs *C9orf72*.**


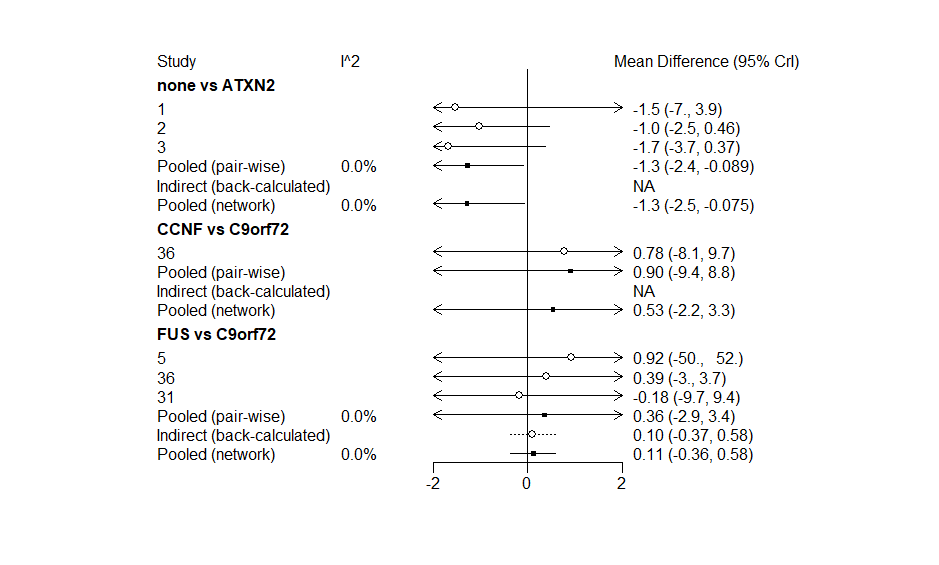


Note:**1,** G. Borghero (2015). **2,** A. Chiò(discovery cohort, 2015). **3,** A. Chiò (validation cohort, 2015). **5,** A. Chiò (2012). **31,** Y. P. Chen (2021). **36**, E. P. McCann (2017)

**Figure 3 forest plot for heterogeneity test for *NEK1 vs C9orf72, none vs C9orf72.***


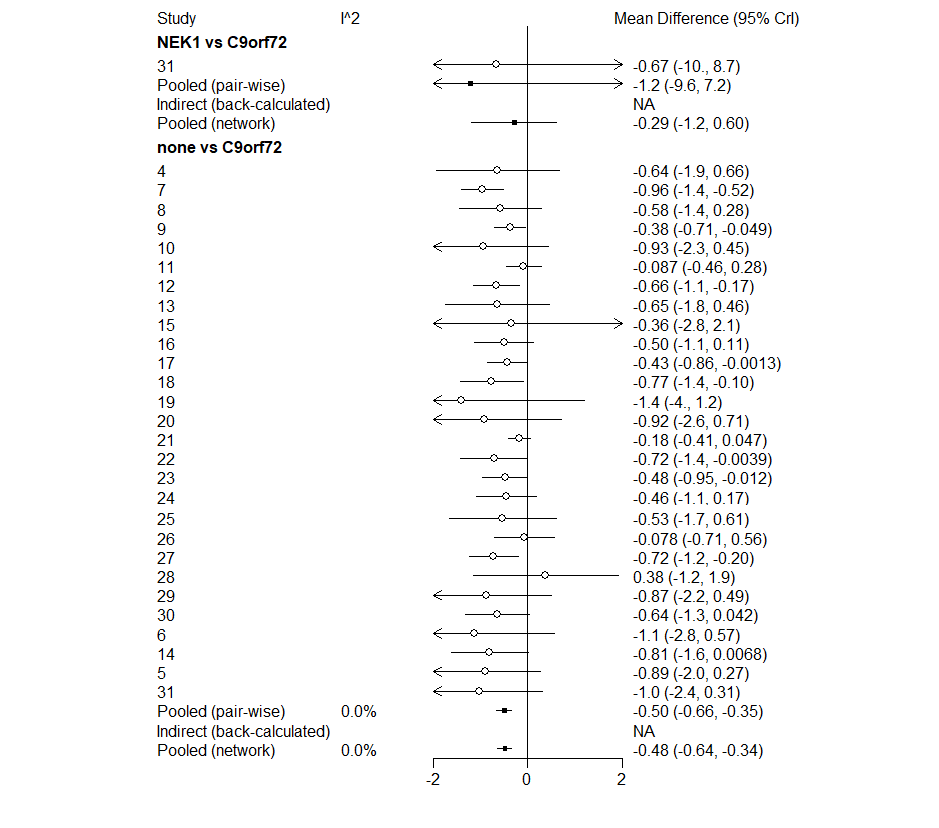


Note: **4,** S. Byrne (2012). **5**, A. Chiò (2012). **6**, S. Lattante (2012). **7.** A. Ratti (2012). **8,** M. Sabatelli (2012). **9,** W. van Rheenen (2012). **10,** S. Debray(sALS cohort,2013). **11,** S. Debray(fALS cohort, 2013). **12,** A. García-Redondo (2012). **13,** D. J. Irwin (2013). **14**, G. Borghero (2014). **15,** K. Van Laere (2014). **16,** A. Calvo (2016).**17,** R. Govaarts (2016). **18,** M. E. Umoh (2016). **19,** T. F. Gendron (2017). **20,** W. Reniers (2017). **21,** A. J. Cammack (2019). **22,**  G. Miltenberger-Miltenyi (2018). **23,** J. Rooney (2019). **24,** F. Trojsi (2019). **25,** M. Benatar (2020). **26,** M. De Schaepdryver (2020). **27,** D. Brand (2021). **28,** U. Kläppe (2021)**. 29,** F. Puentes (2021)**. 30,** S. Millecamps (2012). **31,** Y. P. Chen (2021). **33**, S. Millecamps (2010). **34**, P. Corcia (2012). **36**, E. P. McCann (2017). **37**, W. Liu (2021).

**Figure S4 forest plot for heterogeneity test for *SOD1 vs C9orf72, TARDBP vs C9orf72, TBK1 vs C9orf72.***


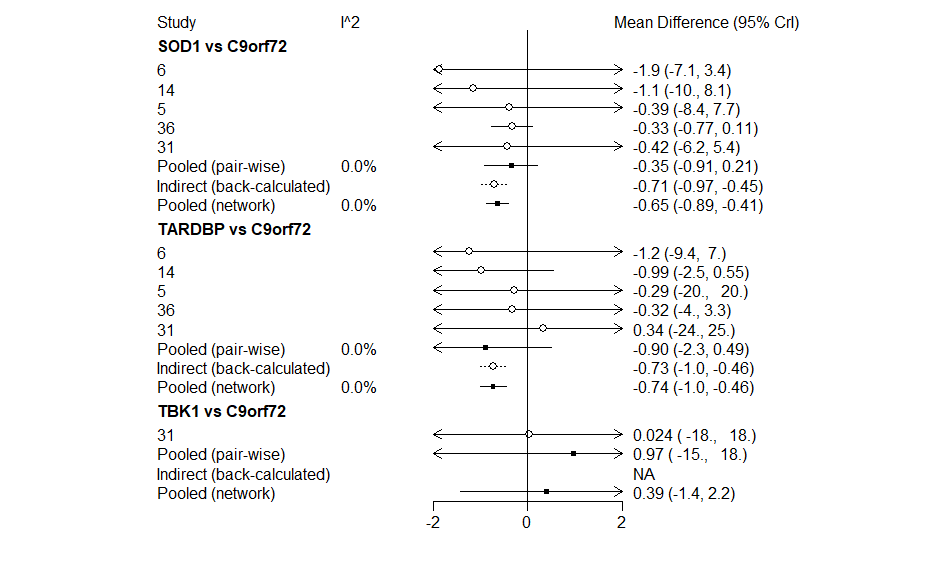


Note: **5**, A. Chiò (2012). **6**, S. Lattante (2012). **14**, G. Borghero (2014). **31,** Y. P. Chen (2021). **36**, E. P. McCann (2017).

**Figure S5 forest plot for heterogeneity test for *UBQLN2 vs C9orf72, FUS vs CCNF, SOD1 vs CCNF, TARDBP vs CCNF.***


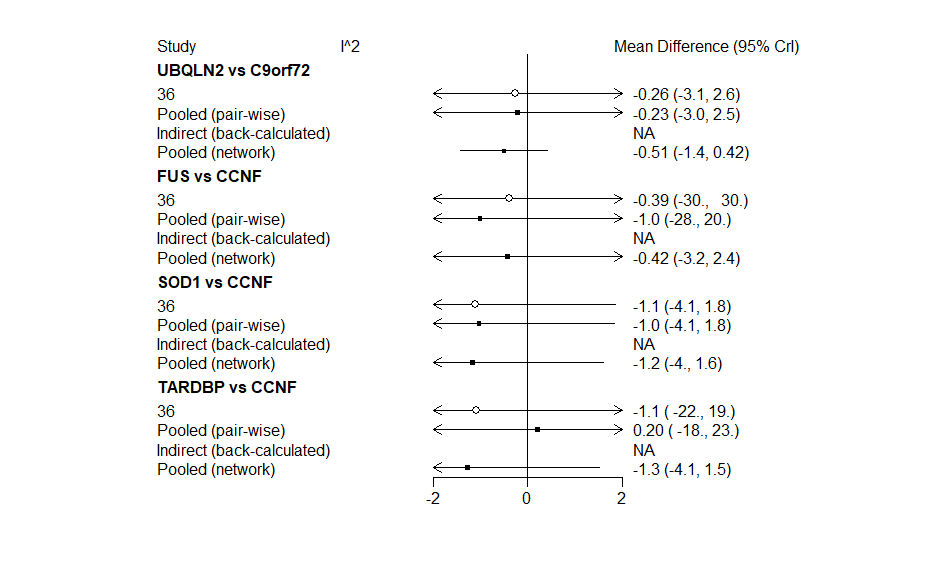


Note: **36**, E. P. McCann (2017).

**Figure S6 forest plot for heterogeneity test for *UBQLN2 vs CCNF, NEK1vs FUS, none vs FUS.***


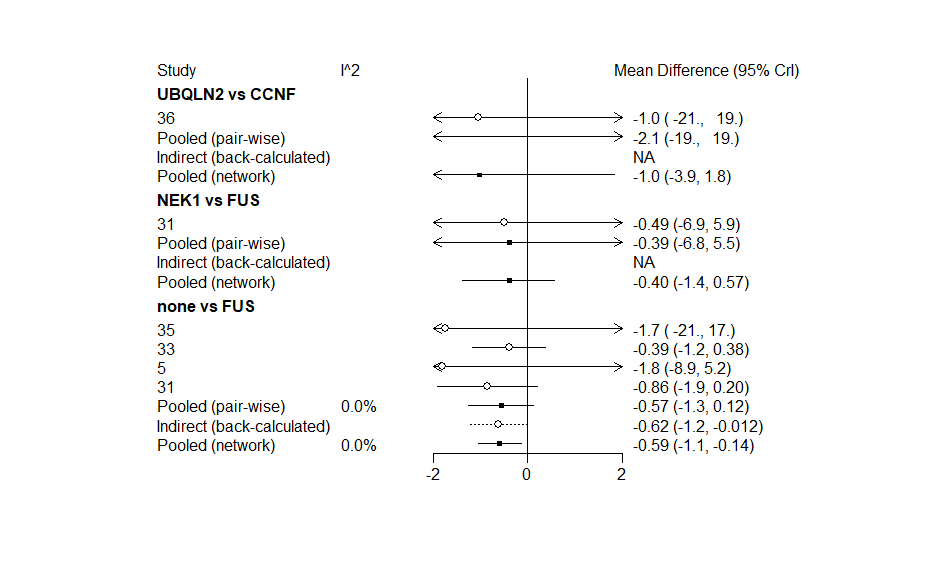


Note: **5**, A. Chiò (2012). **31,** Y. P. Chen (2021). **33**, S. Millecamps (2010). **34**, P. Corcia (2012). **35,** A. Hübers (2012). **36**, E. P. McCann (2017).

**Figure S7 forest plot for heterogeneity test for *SOD1 vs FUS, TARDBP vs FUS, TBK1 vs FUS.***


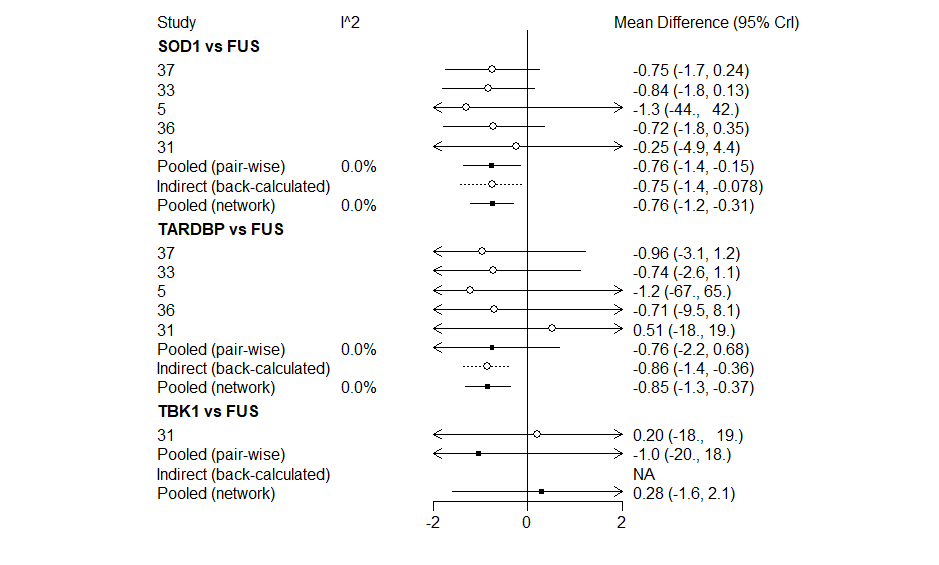


Note: **5**, A. Chiò (2012). **31,** Y. P. Chen (2021). **33**, S. Millecamps (2010). **36**, E. P. McCann (2017). **37**, W. Liu (2021).

**Figure S8 forest plot for heterogeneity test for *UBQLN2 vs FUS, none vs NEK1, SOD1 vs NEK1, TARDBP vs NEK1.***


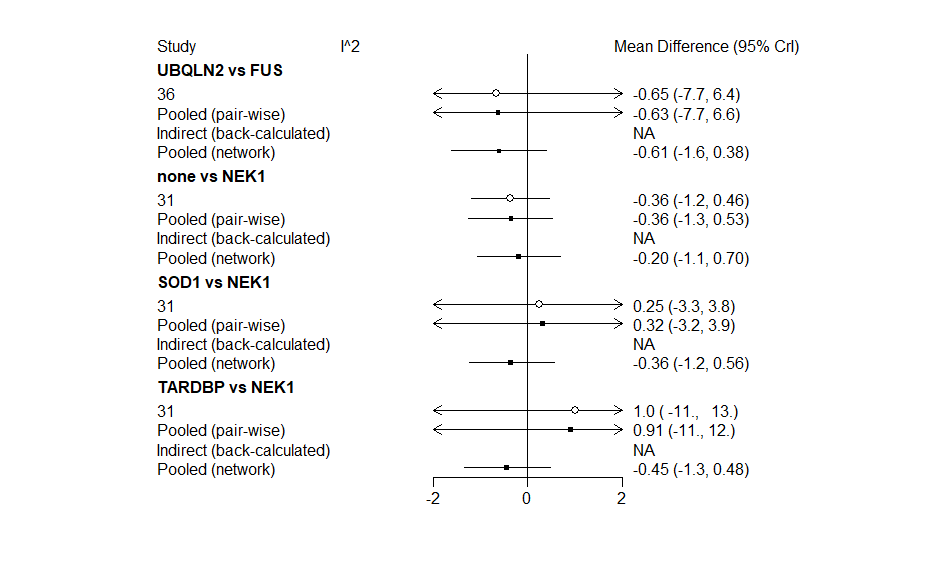


Note: **31,** Y. P. Chen (2021). **36**, E. P. McCann (2017).

**Figure S9 forest plot for heterogeneity test for *TBK1 vs NEK1, SOD1 vs none.***


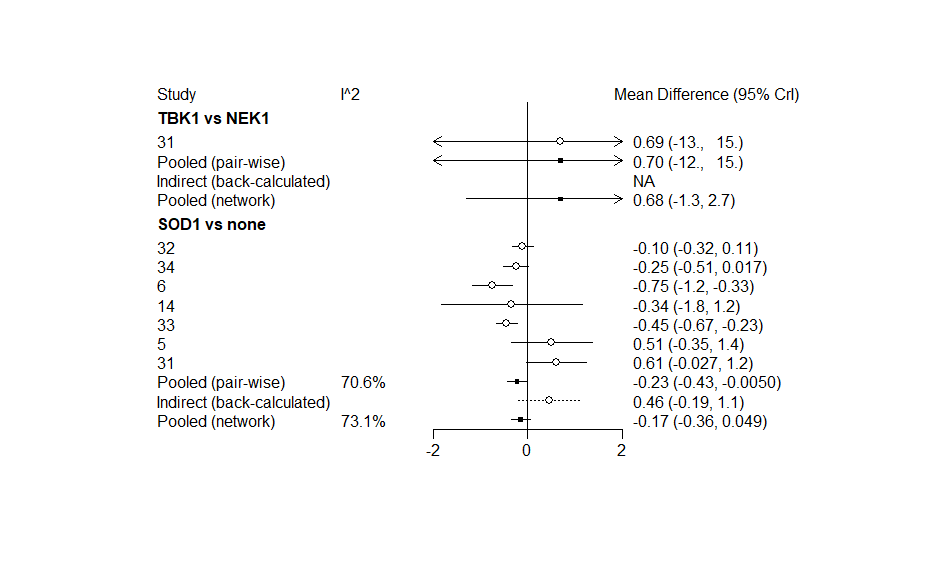


Note: **5**, A. Chiò (2012). **6**, S. Lattante (2012). **14**, G. Borghero (2014). **31,** Y. P. Chen (2021). 32, M. E. Cudkowicz (1997). **33**, S. Millecamps (2010). **34**, P. Corcia (2012).

**Figure S10 forest plot for heterogeneity test for *TARDBP vs none, TBK1 vs none.***


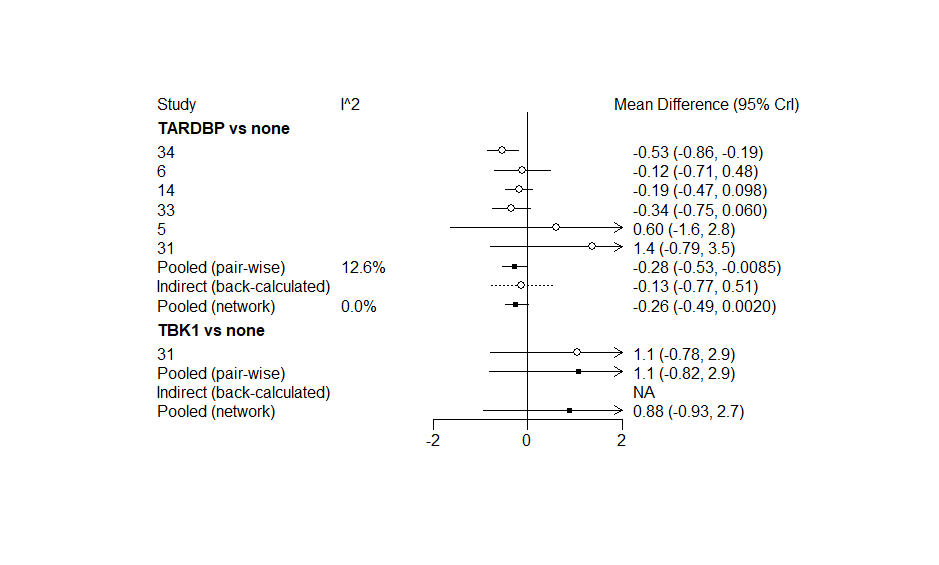


Note: **5**, A. Chiò (2012). **6**, S. Lattante (2012). **14**, G. Borghero (2014). **31,** Y. P. Chen (2021). **33**, S. Millecamps (2010). **34**, P. Corcia (2012).

**Figure S11 forest plot for heterogeneity test for *TARDBP vs SOD1, TBK1 vs SOD1, UBQLN2 vs SOD1.***


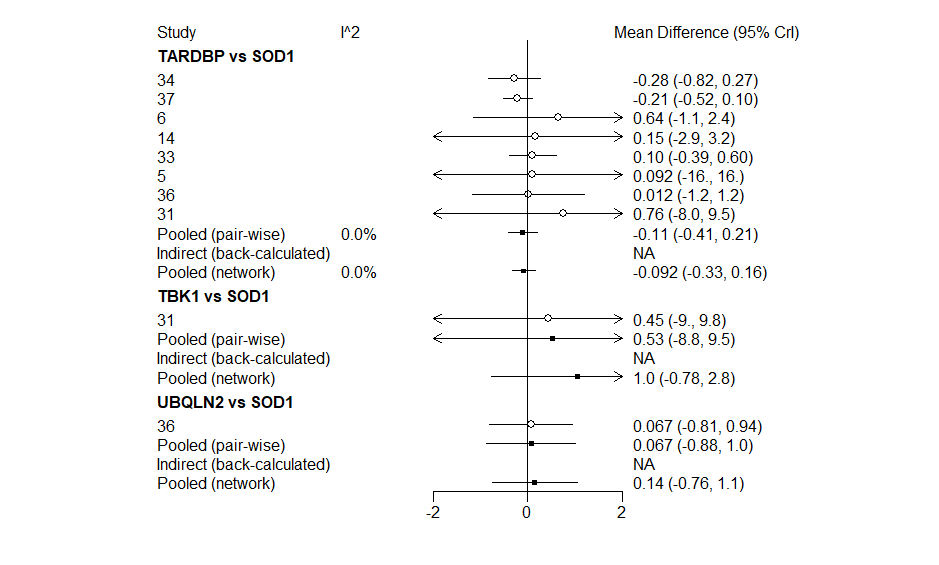


Note: **5**, A. Chiò (2012). **6**, S. Lattante (2012). **14**, G. Borghero (2014). **31,** Y. P. Chen (2021). **33**, S. Millecamps (2010). **34**, P. Corcia (2012). **36**, E. P. McCann (2017). **37**, W. Liu (2021).

**Figure S12 forest plot for heterogeneity test for *TBK1 vs TARDBP, UBQLN2 vs TARDBP.***


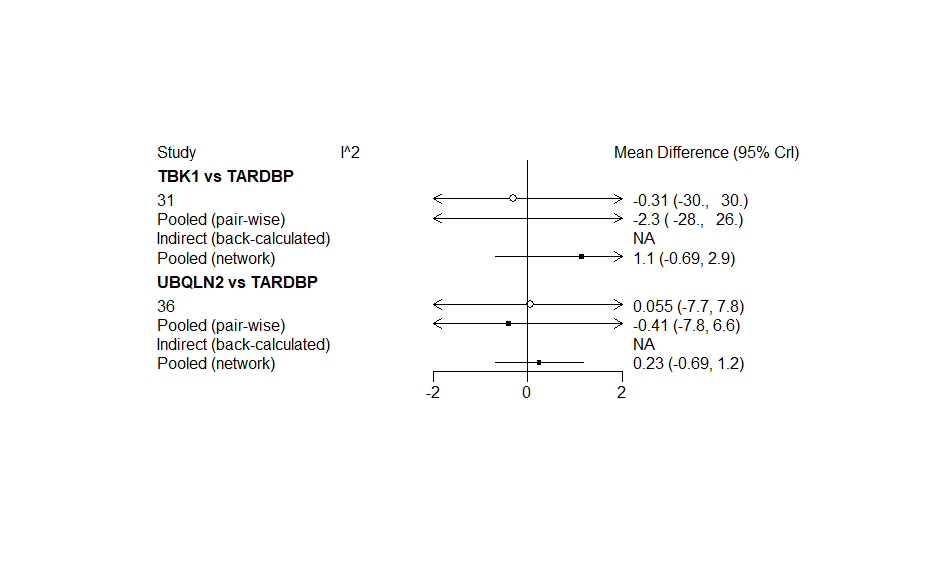
Note: **31**, Y. P. Chen (2021). **36**, E. P. McCann (2017).

**Figure S13** **publication bias for studies reporting *C9orf72* expansion in patients with ALS compared to those without ALS-related mutation.**

Note: Begg's Test

Kendall's Score (P-Q) = 24

Std. Dev. of Score = 50.62

Number of Studies = 28

z = 0.47

Pr > |z| = 0.635

z = 0.45 (continuity corrected)

Pr > |z| = 0.650 (continuity corrected)
